# Mesenchymal stromal cells conditioned by peripheral blood mononuclear cells exert enhanced immunomodulation capacities and alleviate a model of Myasthenia Gravis

**DOI:** 10.1186/s13287-025-04534-9

**Published:** 2025-08-08

**Authors:** Alexandra C. Bayer, Natalia Pinzón, Axel You, Cinthia Bergman, Nadine Dragin, Aurélien Corneau, Frédérique Truffault, Danièle Noël, Christophe Martinaud, Rozen Le Panse, Sonia Berrih-Aknin, Jean-Thomas Vilquin

**Affiliations:** 1 Sorbonne Université, INSERM, Institut de Myologie, GH Pitié-Salpêtrière, Centre de Recherche en Myologie, Paris, F-75013 France; 2 Department of Neurology and Immunobiology, Yale University School of Medicine, New Haven, CT 06520 USA; 3 Institut de Myologie, plateforme MyoData, Paris, F-75013 France; 4 Sorbonne Université, Plateforme de Cytométrie de la Pitié-Salpêtrière (CyPS), Paris, F-75013 France; 5 IRMB, Université de Montpellier, INSERM, Montpellier, F-34295 France; 6 INSERM, Unité des Médicaments de Thérapie Innovante, Centre de Transfusion Sanguine des Armées, Clamart, F-92140 France; 7 Cepheid, Maurens-Scopont, F- 81470 France; 8 Sorbonne Université – INSERM , Institut de Myologie, Centre de Recherche en Myologie, Faculté de Médecine, 105 boulevard de l’Hôpital, Paris Cedex 13, 75013 France

**Keywords:** Mesenchymal stromal cells, Myasthenia gravis, Immunomodulation, Conditioning, γ-Interferon, RNASeq, Mass cytometry, Secretome, Inhibition of proliferation, Humanized mouse model

## Abstract

**Background:**

Mesenchymal Stromal Cells (MSC) possess innate immunomodulatory properties, which can be significantly enhanced through co-culture with peripheral blood mononuclear cells (PBMC), making them attractive tools for the treatment of autoimmune and inflammatory diseases.

**Methods:**

Leveraging a multi-omics approach encompassing RNA sequencing, flow and mass cytometry, secretome analysis, completed by functional evaluations, we investigated the mechanisms underpinning PBMC conditioning of MSC in vitro and their benefits in an animal model of Myasthenia gravis. MSC derived from human adipose tissue were left untreated in resting state (rMSC), conditioned by PBMC (cMSC), or activated by the pro-inflammatory molecule interferon (IFN)-γ (γMSC), then compared for their gene expression profiles, phenotypes and functional capacities.

**Results:**

RNA sequencing identified 244 differentially expressed genes in cMSC compared to rMSC, highlighting key immune mediators such as *CCL2*, *CCL11*,* DPP4*,* ICAM1*,* IL6*,* PDCD1LG2*,* TNFRSF11B*,* TNIP1*,* TNIP3* and *ZC3H12A* and pinpointing genes involved in matrix remodeling, paracrine and autocrine communications. Comparatively, 2089 genes were differentially expressed between rMSC and γMSC, highlighting host defense, anti-viral response, NFκB signaling pathways modulated by IFN-γ. Flow and mass cytometry analyses revealed upregulation of the surface markers CD26, CD54, and CD273 and intracellular molecules IDO1 and PTGS2 in cMSC. In contrast, IFN-γ activation predominantly increased HLA-related markers while also enhancing the homogeneity of the populations. Together, these results underlined the treatment dependence of transcriptomic and phenotypic signatures. Secretome profiling identified 6 categories of modulated proteins, out of which 22 molecules potentially involved in PBMC conditioning and 40 implicated in cMSC-mediated immunomodulation. Functionally, cMSC induced modulation in PBMC subsets, raising the proportions of lymphocyte populations (CD4 Treg, CD8, B memory), underlining the multimodal effect of conditioning. Also, both a direct cell-cell contact and cMSC supernatants significantly suppressed activated T-cell proliferation in vitro. To confirm immunomodulation efficacy in vivo, cMSC were administrated to our humanized mouse model of Myasthenia Gravis and the treatment significantly halved disease severity from 2 weeks post-injection.

**Conclusions:**

This integrative study establishes distinct conditioning signatures, suggests molecular mechanisms, and underscores the therapeutic potential of cMSC, offering a robust framework for advancing cell-based therapies in autoimmune diseases.

**Supplementary Information:**

The online version contains supplementary material available at 10.1186/s13287-025-04534-9.

## Background

Mesenchymal Stromal Cells (MSC) are non-hematopoietic, multipotent progenitor cells that can be isolated from various somatic human adult tissues. Their immunoregulatory capabilities affect both adaptive and innate immunity through cell-cell contact, soluble mediators and the production of extracellular vesicles acting as intercellular messengers [1–4]. They can reduce B and NK cell responses, act on monocyte and macrophage polarization and inhibit dendritic cells maturation [5–7]. MSC also broadly modify T-cell subsets activation and proliferation in vitro and reduce the ability of antigen-presenting cells to exert their role and to produce pro-inflammatory cytokines [7–10]. Their potential to modulate immune responses, suppress inflammation, promote tissue repair, and restore immune homeostasis suggested their potential therapeutic usefulness for the treatment of several immune-mediated or autoimmune diseases such as systemic lupus erythematosus, multiple sclerosis, rheumatoid arthritis, Sjögren’s syndrome [11–16]. Indeed, MSC can be prepared in large numbers from adult adipose tissue and validated for clinical use along international guidelines [17–19], and their use as immunomodulation agents has been investigated in dozens of trials without toxicity, but frequently with low efficacy. Recent evidence has reshaped the methodology and refined the indications for their large-scale use [13, 20–22] and promising clinical results were achieved [20, 23, 24]. There is agreement that conditioning, or priming of the cells before use will improve their biological and immunomodulatory capacities [25–33]. MSC can be conditioned by peripheral blood mononuclear cells (PBMC) or primed by factors such as IFN-γ, TNF-α or other interleukins [3, 21, 28, 31, 33, 34]. The molecular priming usually requires supra-physiological doses of agents and acts through specific paths [33]. The optimal conditioning agent is not yet determined, as currently used cytokines may have broad activities and potentially increase the immunogenicity of the cells [35, 36]. Conditioning MSC with PBMC may appear more physiological and offer greater specificity and could represent a more multimodal fashion, however, its mechanisms are still largely unknown.

*Myasthenia Gravis* (MG) is a well-defined prototypical autoimmune disease mediated by pathogenic antibodies (Ab) targeting mainly the acetylcholine receptor (AChR) at the neuromuscular endplate [37]. The disease is associated with severe defects in immune regulation, chronic cell activation, and inflammation [38–40]. The thymus is considered the effector organ in early onset forms of MG associated with anti-AChR Ab, it is highly inflammatory and characterized by the abnormal recruitment of peripheral B cells and ectopic germinal center development leading to the production of autoreactive B cells [39–41]. In this indication, the clinical status of patients may be improved by thymectomy [42]. MG leads to abnormal fatigability and can be life-threatening. Treatments palliate the decrease in AChR (acetylcholinesterase inhibitors), reduce the overall immune reactions and inflammation (glucocorticoids and immunosuppressant) or target specific compartments (new biotherapies directed against B or T cell subtypes, complement, Ab recycling), but despite considerable progresses there is still no curative treatment for this invalidating disease [43, 44]. Additionally, life-long medications, especially glucocorticoids, have serious deleterious side-effects affecting several tissues and organs. Both disease physiopathology and the remaining medical needs promote MG as a good prototypic model for exploring the efficacy of conditioned MSC in an autoimmune disease. In this aim, we previously established an humanized model, called NSG-MG, based on grafting pieces of MG thymus into immunodeficient mice (NOD-SCID-Gamma, NSG). These thymic fragments induced most animals to harbor Ab and to develop clinical symptoms [34]. Using MSC grown in fetal bovine serum (FBS), we previously compared the effect of human resting MSC (rMSC) with MSC conditioned by PBMC (cMSC) in NSG-MG mice and we observed that cMSC were superior to rMSC for their therapeutic effects, through the regulation of CD55 and of TNFα family ligands in vivo [34]. Beneficial effects of MSC in alternative models of MG were also observed by others using syngeneic MSC in rats [45] or human MSC in mice [46].

The use of cells as therapeutic agents, however, requires this product to be as robustly characterized as possible, and its main mechanisms of activation unveiled. To facilitate future clinical translations for the use of cMSC, in this study we utilized human MSC initially prepared by a cell therapy laboratory (Centre de Transfusion Sanguine des Armées, Clamart), and using human platelet lysate (hPL) instead of xenogenic FBS. Upon conditioning of MSC by PBMC, (a) we assessed the modifications in MSC gene expression levels induced by the reciprocal interactions between MSC and PBMC in vitro, as compared to the effect of IFN-γ used as a control for priming. (b) We evaluated the modifications of phenotypical markers in MSC and the modulation of PBMC subsets upon their interactions. (c) We described the differential secretomes produced by rMSC, PBMC, MSC-PBMC coculture and cMSC. (d) We quantified the functional efficacy of cMSC *in* vitro through inhibition of cell proliferation and polarization of populations, and we confirmed their therapeutic benefit in our preclinical MG model. Finally, we proposed and validated markers for potency, and we explored the mechanisms deployed upon conditioning.

## Methods

### Human samples

#### Mesenchymal stromal cells

Human adipose-derived MSC were isolated from patients undergoing liposuction as *res nullius* upon written consent and anonymization, according to the French laws and regulations. The cells were processed in the human cell therapy laboratory. Donors of M1, M2, M3, M4 were women aged of 57, 39, 49 and 42 years, respectively. Adipose tissues were washed 3 times with PBS (Biological Industries, Kibbutz Beit-Haemek, Israel) and digested in type I Collagenase (Sigma Aldrich, Lyon, France) for 1 h at 37 °C then centrifuged (200 g). The stromal vascular fractions were washed with αMEM (Biological Industries) supplemented with 10% human albumin (LFB, Les Ulis, France). Isolated mononuclear cells were seeded between 10 000 and 20 000 cells/cm^2^ and cultured at 37 °C in 5% CO_2_ in a medium containing αMEM, 2UI/mL Sodium Heparin (Sanofi, Paris, France) and 5% human platelet lysate (hPL) obtained from the French Army blood transfusion center (CTSA, Clamart, France) as previously described [47]. When reaching 80–90% of confluence, MSC were trypsinized (Trypzean^®^, Sigma-Aldrich) and frozen in αMEM supplemented with 10% human albumin and 10% DMSO (Sigma).

#### Peripheral blood mononuclear cells

PBMC used for MSC conditioning and CFSE assay were obtained from venous blood from healthy volunteer donors (French Blood Establishment, EFS, Rungis, France) using the Lymphoprep density gradient centrifugation protocol (Axis-Shield, Oslo, Norway), suspended in FBS (HyClone, Utah, USA) supplemented with 10% DMSO and stored frozen at -150 °C. Donors (P1 - P5) were 20–64 years old.

#### MG thymus

AChR^+^MG thymic fragments required to establish the humanized mouse model were obtained from patients undergoing thymectomy (Paris, France) who gave written informed consent for the use of *res nullius* samples obtained during surgeries and were anonymized. Two MG female thymuses (16 and 21 years old), with no thymoma, were included.

### Cell cultures

#### MSC thawing, culture and expansion

Frozen MSC provided by the cell therapy laboratory were thawed and washed in αMEM (Gibco), seeded at a density of 4,000 to 5,000 cells/cm^2^ in non-coated plastic vessels (Corning, Boulogne-Billancourt, France) and grown in αMEM supplemented with penicillin/streptomycin (Gibco), 5% hPL (MacoPharma, Tourcoing, France) and 2U/mL of heparin (Sigma Aldrich). Adherent cells were cultured with regular change of medium and passaged once using 0.05% Trypsin/EDTA (Gibco). They were harvested at the end of passage 1 (80% confluence) and frozen at a concentration of 1 × 10^6^cells/mL in αMEM supplemented with 20% FBS and 10% DMSO.

#### MSC conditioning or priming, production of supernatants and conditioned media

In vitro, MSC conditioning consisted of a 3-day coculture with allogeneic PBMC and priming consisted in 2 days activation with IFN-γ. Briefly, passage 2 MSC were thawed and seeded at density of 4000 cells/cm^2^ for 4 days, then plated into 6-well plates at 4000 cells/cm^2^ in growth medium. Four days later, as MSC reached a density between 30 000 and 40 000 cells/cm^2^, the wells were equipped with Transwell membrane cell culture inserts (1 μm pore size, Becton Dickinson, Rungis, France) and either thawed allogeneic PBMC (2,5:1 PBMC to MSC ratio) were added to produce conditioned MSC (cMSC), or medium alone to produce resting MSC (rMSC). In parallel, 6-well plates of MSC grown as above were incubated with IFN-γ for 48 h (500 UI/ml, Biotechne, R&D Systems) to produce γMSC.

After treatment, MSC and PBMC cells were harvested. The culture and coculture supernatants were collected, centrifuged (650 g, 10 min at 4 °C) and frozen. The PBMC were counted and frozen. The rMSC, cMSC and γMSC were harvested by trypsination, counted and splitted for uses in different experimental set-ups. One fraction was frozen for future mass cytometry analyses and another one for RNA extraction. One fraction was used for immediate phenotypical analysis by flow cytometry. And one fraction was seeded at 50,000 cells/cm^2^ for production of rMSC, cMSC and γMSC supernatants and cells for 3 days (D3-cMSC), which were then kept frozen as above. Three to 4 MSC cultures were each combined with 3 to 5 PBMC donors.

#### Flow cytometry phenotyping

To establish the differential phenotypic signatures of cMSC and γMSC by flow cytometry, 23 samples were used (4 rMSC, 15 cMSC, 4 γMSC) to compare the expression of markers found dysregulated in our study or in the literature. Resting or conditioned cells were washed with PBS and incubated with monoclonal fluorochrome-conjugated Ab (30 min at 4 °C). These Ab were selected from literature survey [48, 49], our previous researches [50, 51], and markers identified through the RNASeq study. Control isotypes were used for all types of immunoglobulins (Ig), and for all fluorochromes. At least 50,000 cells were acquired using a BD Canto II cytometer (BD Biosciences) or a CytoFlex S (Beckman Coulter, Villepinte, France). Data were analyzed with FlowJo software version 10.7.1 (Tree star, Olten, Switzerland). The geometric mean fluorescence intensity (MFI) for each marker was compared among conditions and to the corresponding isotype control. The whole Ab list is presented in Supplemental Table 1. Comparisons among independent MSC cultures were performed using ANOVA. Statistical significance is recognized at *p* <.05.

### Mass cytometry (CyTOF) phenotyping of MSC and PBMC

#### Design of the panel of cytof markers

To define specific groups of sub-populations and their changes among rMSC, cMSC or γMSC, or to validate their potential homogeneities, we conducted single-cell mass cytometry analysis of 30 cell preparations. MSC characterization was done using a 27 monoclonal anti-human metal-tagged Ab panel that included cell surface, cytoplasmic, and nuclear targets as detailed in Supplemental Table 1. Surface markers were selected after evaluation of their expression by MSC, with or without conditioning, by flow cytometry. We retained the markers that showed important changes in MFI between conditions, and/or heterogeneous histograms (by comparing their robust coefficient of variation). Three intracellular markers (PTGS2, IDO1 and the proliferation-associated nuclear protein ki-67) were added to the panel as they are considered important actors of immunomodulation. Metal-Ab matches were configured by the CyTOF platform facility (Cytometry Pitié-Salpêtrière, CyPS), using the values of geometric mean established by flow cytometry. Ab were either purchased pre-conjugated from Fluidigm or purchased purified and conjugated in-house using MaxPar X8 Polymer Kits (Fluidigm, Les Ulis, France) according to the manufacturer’s instructions.

#### Design of dedicated MSC surface barcoding

Barcoding allows gathering and analyzing of multiple samples within the same tube to reduce the technical variability of the results. As the classical barcoding method requires tough permeabilization protocols impacting the stability of extracellular antigens in our settings, we designed a gentler barcoding procedure based on CD90 expression, a well-known hallmark of MSC. To limit steric hindrance, clones recognizing different epitopes of the CD90 antigen were used and coupled to distinct metal isotopes as defined in Supplemental Table 1. A unique combination of 3 isotopes was attributed to each sample and allowed the study of 15 samples in the same tube.

#### Barcoding and labeling of the cells

The methodology was followed according to Dzangué-Tchoupou et al. [52]. Briefly, cells were thawed, washed in PBS (Maxpar, Fluidigm), suspended at 1 × 10^6^ cells/ml and incubated with 5µM of Cisplatin (5 min at room temperature (RT), Fluidigm). Then cells were washed in staining buffer (SB, Fluidigm). The 30 samples were splitted in 2 series of 15 samples for barcoding. Each sample was barcoded with a unique combination of 3 anti-CD90 Ab tagged with different isotopes and incubated (37 °C, 40 min). After individual barcoding, samples were washed and gathered in their corresponding series and stained with Ab targeting cell surface antigens (RT, 30 min). After washing, cells were fixed and permeabilized using the BD Cytofix/CytoPerm kit (BD Bioscience) (RT, 60 min), and incubated with Ab directed against intracellular antigens (1 h, RT). After washing, cells were fixed with paraformaldehyde (PAF) 1,6% (Pierce, Thermo-Fisher) in SB (10 min, RT), washed and labeled with the DNA intercalator Iridium-125 (0.1%, 4 °C, overnight, Fluidigm) in MaxPar Fix and Perm buffer (Fluidigm) and frozen (-80 °C) until acquisition. Then, cells were washed extensively in SB, resuspended with standardization/normalization Eq. 4 Elements beads. 2 × 10^5^ to 3 × 10^5^ cells were acquired on the Helios 2 Mass Cytometer (Fluidigm). The 2 tubes were acquired successively in the same operating day.

#### Debarcoding and analysis of labeled MSC

FlowJo software was used for data cleaning and debarcoding. After beads removal (Ir193^+^/Ce140^+^), singlets (191Ir^+^/193Ir^+^) and viable MSC (CD105^+^/195Pt^-^) were gated manually. Boolean gating based on different metal-tagged CD90 was performed to obtain sample separation. Equal event sampling was selected, using 10^4^ events per sample (the lowest common denominator across all). Non-supervised and supervised analysis were performed using Omiq software (www.omiq.ai, California, USA). First, we performed dimensionality reduction based on all markers (except for CD90, used for barcoding) and using the Optimized t-distributed stochastic neighbor embedding tool (Opt-SNE) (Max Iterations: 7500, Perplexity: 40 and Theta: 0,5), followed by single-cell data categorization/clusterization into ostensible cellular populations using ClusterX tool (Alpha: 0,001). The feature characteristics and abundance patterns of each cluster were analyzed using clustered heatmap.

#### Barcoding and labeling of PBMC (MDIPA™ assay)

To address the functional changes induced in the PBMC populations during the conditioning step, we used the Maxpar Direct Immune Profiling Assay™ (Fluidigm^®^), a ready-to-use CyTOF panel, to profile 37 immune cell subsets. PBMC harvested and frozen after the coculture step (*n* = 15 to 16) were thawed, washed with PBS and incubated in 250U of Nuclease (Pierce, Thermo-Fisher, 37 °C 30 min). To avoid biases due to donor intrinsic variability, samples of the different PBMC placed in coculture were compared to counterpart samples of the same donors but grown alone in culture media in wells lacking MSC (*n* = 4). We implemented the barcoding of samples based on CD45 to reduce the technical and batch effects. 1 × 10^6^ cells of each sample were barcoded using the labeling of CD45 tagged to the different isotopes Cd106, Cd111, Cd113, Cd116 (Clone HI30; Fluidigm) and pooled groups of 4 samples in a single tube keeping a maximum of 3 × 10^6^ cells. Fc receptors of barcoded samples were blocked using Human TruStain FcX (Biolegend, Paris, France) and incubated for 10 min at RT. Cells were then stained using the Maxpar Direct Immune Profiling Assay™ (Fluidigm). Briefly, FcR-blocked PBMC were diluted in SB and stained. After 30-min RT incubation, the cells were washed twice in SB, followed by fixation in 1.6% PAF for 10 min. Then, the cells were spun to a pellet which was suspended in 1 ml of the 125 nM Cell-ID Intercalator-Ir (4 °C, overnight, Fluidigm). Immediately prior to acquisition on Helios XL (Fluidigm), samples were washed, counted and diluted with PBS mixed with Eq. 4 Element beads at the CyPS core facility. A maximum of events was acquired per sample. Mass cytometry standard files produced by the Helios were normalized using the CyTOF software v.6.7.1014. This method normalizes the data to a global standard determined for each log of EQ beads.

#### Debarcoding and analysis of PBMC

Debarcoding of PBMC was done as described for MSC and individual FCS files generated were analyzed using Maxpar Pathsetter, an automated analysis system powered by GemStone™ 2.0.41 (Verity Software House, Topsham, ME). This system is integrated with dimensionality-reduction mapping known as Cauchy Enhanced Nearest-neighbor Stochastic Embedding (Cen-se′™), which generates a visual display of high-dimensional data labeled with the major cell populations. Cell subset frequencies were compiled in tables and were used for statistical analysis.

### Gene expression analysis

#### RNA Preparation

A RNAseq study was performed to compare exclusively the levels of gene expressions by MSC between treatments. Total RNA from 1.5 × 10^6^– 2 × 10^6^ rMSC (*n* = 4), cMSC (*n* = 13) and γMSC (*n* = 3) cells was extracted using the mirVana miRNA Isolation Kit (Thermo-Fisher) and used for RNASeq study and qRT-PCR validations (see below). RNA concentration and purity were determined using NanoDrop ONE (Thermo-Fisher). All samples presented ratios from 1.9 to 2.20 for 260/280 nm and 260/230 nm. The RNA quality was assessed on gel using ARN FlashGel™ System (Lonza Bioscience, Basel, Switzerland). In samples used for RNAseq purposes (rMSC (*n* = 3), cMSC (*n* = 9) and γMSC (*n* = 3)), RNA integrity was further assessed with a Bioanalyzer 2100 (Agilent Technologies, California, USA), RNA integrity number was over 9. RNA was reverse-transcribed using a concentration of 500ng/µL for 1 h at 42 °C using AMV (Roche, Merck, Fontenay-sous-Bois, France) with oligo-dT (Thermo-Fisher).

#### RNAseq analysis

RNA sequencing approach was carried out at the Genom’ic Core Facility at the Institut Cochin, University of Paris Descartes. Briefly, ≈ 1 µg of total RNA was isolated from cells and depleted of rRNA with the low Input RiboMinus Eukaryote System v2 (Ambion, Thermo-Fisher). The depleted RNAs were used to generate cDNA libraries according to the manufacturer’s protocol (Ion total RNAseq kit V2, Thermo-Fisher). The sequencing was performed on NextSeq 500 from Illumina (Illumina, California, USA) using paired-end 150–base pair reads (at least 10 million reads per sample). After quality control of the run, adaptor and low-quality trimming and removal of contaminants, the reads were splice-aware mapped to a reference genome including annotations (GRCh38.p13, Ensembl release 101) using the STAR aligner v2.7.6a. Gene-level quantification was done with RSEM (v1.3.1). Raw counts were normalized, genes with low counts (≤ 10 in at least 3 samples) were discarded. Analysis for differential expression between conditions, accounting for variations due to batch effect (Batch + Condition), was done fitting to a negative binomial generalized model implemented in the DESeq2 algorithm (R package, version 1.26.0). Genes with adjusted pvalue < 0.05 were regarded as differentially expressed genes. General quality controls were performed, that showed low duplicated reads, excellent mapping percentages and complexity of the libraries (using Fastqc 0.11.9, picard 2.23.9, preseq 3.1.1, dupradar 1.18.0). The data have been deposited in the European Nucleotide Archive (ENA) at EMBL-EBI under accession number PRJEB77871 (ERP162200).

Hierarchical clustering of the top 80 most significant differentially expressed genes was performed on the normalized matrix. Distance between features was measured by (1 - Spearman correlation coefficient) and clustering was performed using the Ward.D2 method.

#### Annotation/research of cell markers

Differentially expressed genes were categorized according to their annotation on GO ontology at the release from 2021-09-01. Genes annotated as “integral components of membrane” (accession GO:0016021) (excluding genes products in this term that are also categorized as endoplasmic reticulum and respiratory chain components), as “catalytic activity” (accession GO:0003824), and as “extracellular space” (accession GO:0005615) were further studied.

#### Pathways analysis

ClusterProfiler v4.2.0 R package was used for all enrichment analysis. Gene Set Enrichment Analysis [53] was performed on the entire gene set, with a minimum size of gene set accepted of 10. All collections available in the Molecular Signatures Database (http://www.gsea-msigdb.org/gsea/msigdb/collections.jsp) were queried. Redundant terms were manually removed and most interesting ones according to their biological activity were further studied.

#### Primer design and validation

Gene expression modulation was confirmed by qRT-PCR using a panel of candidate genes deduced from the RNAseq study supplemented with *PDCD1LG2* which emerged from a previous cytometric study. The primer sets were obtained using primer3.ut.ee and setting the following conditions: product size between 150 and 250 bp, max melting temperature (Tm) difference of 5, and an optimal primer Tm of 60 °C. Primer sequences and details are shown in Supplemental Table 2. One µg of RNA was reverse-transcribed for 1 h at 42 °C using AMV (Roche, Merck) with oligo-dT (Thermo-Fisher). PCR reactions were carried out in duplicates using the LightCycler 480 SYBR Green Master Mix on the LightCycler^®^ 480 System and following manufacturer’s instruction (Roche Diagnostics, Meylan, France).

#### QPCR analysis

A total of 20 samples were assessed including rMSC (*n* = 4), cMSC (*n* = 13) and γ-MSC (*n* = 3) in duplicates. The normalized expression levels were calculated according to the ΔΔCt method. Briefly, ΔCt values were determined as the difference between the cycle threshold (Ct) value of the gene of interest and the mean Ct value of the housekeeping gene GAPDH. Expression was normalized as specified in the figure legends. A One-way ANOVA test was performed to evaluate the significance of MSC gene expression modulation induced by the different treatments.

### PBMC proliferation Inhibition

We challenged and compared the functional efficacy of rMSC, cMSC, γMSC themselves (direct cell-cell contact) and of their secretomes to inhibit the proliferation of activated T cells using the CFSE assay. Briefly, healthy PBMC were labeled with CFSE using CellTrace Cell Proliferation Kit (Invitrogen, Thermo-Fisher, 20 min at 37 °C), re-suspended in complete media supplemented with beads covalently coupled with anti-CD3, anti-CD28 Ab according to manufacturer instructions (Dynabeads, Gibco). They were transferred to wells containing rMSC, cMSC or γMSC in 1:10 MSC/PBMC ratio or to wells containing MSC supernatants, in a concentration of 1 × 10^6^ PBMC/mL. Non-activated PBMC and activated PBMC incubated without MSC or their supernatants were used as controls. After 72 h of culture, the percentages of CD4^+^ and/or CD8^+^ proliferating cells out of the total CD45^+^ live cells were determined using a flow cytometer by monitoring the CFSE fluorescent values. Briefly, PBMC were harvested from each well, washed with PBS and stained with CD45-eFluor450, CD4-PE, CD8-APC (Biolegend) and Fixable Near-IR Live/Dead Staining kit (Invitrogen). To assess the role of soluble DPP4 (i.e. CD26) in the blockade of proliferation, saxagliptin (hydrochloride salt, Biotechne, R&D System), a potent inhibitor of this enzyme, was incubated in the conditioned media at 10^− 6^ M for 3 days, i.e. the whole duration of the proliferation assay. Twenty conditions were tested (4 MSC cultures of either resting, activated by IFNγ, or conditioned by 3 PBMC donors).

### Proteome sample preparation and analysis

To identify proteins involved in MSC conditioning or immunomodulation, we conducted a secretome analysis using the proximity extension assay developed by Olink^®^. Complete culture medium (*n* = 2), supernatants from PBMC alone (*n* = 3), rMSC alone (*n* = 4), PBMC-MSC cocultures (*n* = 4), and D3-cMSC (*n* = 4) were harvested after cells incubation for 72 h. Supernatants were centrifuged (650 g, 10 min, 4 °C) before sampling and aliquots were kept at -80 °C. Samples were analyzed simultaneously for 609 unique protein biomarkers on seven pre-designed Proseek Multiplex^®^ immunoassay panels: Cardiometabolic, Cardiovascular II, Cardiovascular III, Development, Immuno-Response, Immuno-Oncology, Neurology (Olink Proteomics, Uppsala, Sweden). Processing, output data quality check and normalization were performed by Olink Proteomics. Raw data were normalized to the company’s internal controls and delivered as Normalized Protein eXpression (NPX) values, a relative protein quantification arbitrary unit from Olink expressed on log2 scale. Validation data and limits of detection are available at the manufacturer’s webpage (http://www.olink.com). Data values below LOD were removed from the dataset and proteins with > 50% missing values were also excluded. Analysis of differentially secreted proteins (padj value < 0.01) was performed using Olink^®^ Insights Stat Analysis app (www.olink.com). Data visualization by PCA plots and heatmaps was done taking into account the 177 differentially secreted proteins and using *Clustvis* [54], a web tool for visualizing clustering of multivariate data. Volcano plots showing potentially activating and immunomodulating proteins were prepared using all measured proteins and the *VolcanoseR* web application [55]. The Table 1 presents the different categories of selected proteins (consumed, activating, inhibited, immunomodulatory) in association with their measurements in the different secretomes according to the Olink methodology.

**Table 1:** Proteins detected by secretome analysis

**a) Proteins consumed by the MSC during culture**										
**Consumed**		**Medium**	**PBMC**	**rMSC**	**Coculture**	**D3 cMSC**	**Med-PBMC**	**Med-rMSC**	**Med-Cocult**	**Med-D3 cMSC**
EGF		**5**,**08**	5,33	2,33	2,06	1,65	-0,26	2,75	3,01	3,42
PDGF - A		**5**,**95**	6,05	3,24	3,28	2,78	-0,11	2,71	2,67	3,16
PDGF - B		**6**,**17**	6,33	3,37	3,52	3,00	-0,16	2,80	2,65	3,17
TCN2		**8**,**76**	8,97	6,72	7,01	6,51	-0,21	2,04	1,76	2,26
STIP1		**5**,**17**	5,58	3,67	3,61	3,07	-0,41	1,50	1,56	2,10
SRC		**5**,**29**	5,60	4,17	4,59	4,83	-0,30	1,13	0,71	0,46
GLO1		**3**,**03**	2,20	2,27	2,40	2,55	0,83	0,76	0,63	0,48
**b) Proteins potentially participating to the conditioning**										
**Conditioning**		**Medium**	**PBMC**	**rMSC**	**Coculture**	**D3 cMSC**	**Coc-Med**	**Coc-PBMC**	**Coc-rMSC**	**Coc-D3 cMSC**
MMP7		5,20	6,35	5,18	**6**,**88**	5,37	1,68	0,54	1,70	1,52
CCL24		5,54	8,20	5,46	**9**,**06**	4,78	3,52	0,86	3,61	4,29
TNF		0,55	2,43	0,90	**2**,**08**	0,86	1,52	-0,35	1,18	1,21
MPO		2,52	3,84	2,46	**4**,**02**	2,52	1,51	0,18	1,56	1,50
CCL4		1,10	4,77	1,54	**4**,**25**	1,02	3,15	-0,52	2,71	3,24
CD5		1,04	2,32	1,19	**2**,**29**	1,28	1,25	-0,02	1,11	1,01
TR-AP		4,02	5,87	3,70	**5**,**39**	3,54	1,37	-0,48	1,69	1,85
AZU1		2,50	4,96	2,50	**5**,**10**	2,70	2,60	0,14	2,60	2,40
GZMA		1,03	1,93	1,36	**2**,**12**	1,54	1,09	0,19	0,76	0,59
IL-1ra		0,08	3,48	0,52	**4**,**20**	0,72	4,12	0,71	3,68	3,48
LOX-1		1,01	2,88	0,86	**2**,**95**	0,99	1,94	0,07	2,09	1,95
CTSS		1,51	2,57	2,10	**3**,**30**	2,34	1,79	0,73	1,20	0,96
TNF-R2		5,08	5,60	4,97	**5**,**55**	4,91	0,47	-0,05	0,58	0,64
CCL3		3,28	6,42	2,86	**5**,**13**	2,43	1,85	-1,28	2,28	2,70
IL16		0,91	1,27	0,81	**1**,**49**	0,95	0,58	0,22	0,68	0,54
GNLY		3,40	4,49	3,51	**5**,**23**	2,76	1,83	0,74	1,72	2,47
CXCL16		4,60	5,29	4,70	**5**,**59**	4,55	0,98	0,29	0,88	1,04
CHI3L1		5,49	6,10	5,66	**6**,**44**	5,98	0,95	0,34	0,78	0,46
KYNU		1,45	1,50	1,16	**1**,**81**	1,43	0,36	0,31	0,64	0,37
CCL17		4,92	5,30	5,24	**5**,**82**	4,42	0,90	0,52	0,57	1,40
MSR1		1,12	1,39	1,22	**1**,**56**	1,22	0,44	0,17	0,34	0,34
STC1		0,61	0,86	2,83	**4**,**04**	3,49	3,44	3,18	1,21	0,55
**c) Proteins inhibited by the conditioning**										
**Inhibited**	**Medium**		**PBMC**	**rMSC**	**Coculture**	**D3 cMSC**	**D3 cMSC-Med**	**D3 cMSC-PBMC**	**D3 cMSC-rMSC**	**D3 cMSC-Coc**
TIMP1	9,87		10,23	10,82	10,66	**10**,**01**	0,14	-0,22	-0,81	-0,65
CCL17	4,92		5,30	5,24	5,82	**4**,**42**	-0,50	-0,88	-0,83	-1,40
SAA4	8,43		8,47	8,44	8,49	**8**,**20**	-0,23	-0,28	-0,24	-0,30
CCL19	2,47		2,69	2,72	2,76	**2**,**02**	-0,45	-0,67	-0,70	-0,75
PAI	8,66		8,84	9,43	9,14	**8**,**81**	0,14	-0,04	-0,62	-0,33
CXCL11	2,16		1,69	2,02	2,19	**1**,**12**	-1,04	-0,57	-0,90	-1,07
SNAP29	8,16		8,43	8,13	8,19	**6**,**17**	-2,00	-2,27	-1,96	-2,03
SPINK1	2,71		2,92	2,67	2,94	**1**,**72**	-0,99	-1,20	-0,95	-1,21
TNFRSF13B	3,54		3,54	3,43	3,64	**3**,**01**	-0,54	-0,53	-0,42	-0,64
ADM	1,55		1,01	4,79	5,58	**2**,**68**	1,13	1,67	-2,11	-2,90
**d) Proteins potentially involved in the immunomodulatory capacity of cMSC**										
**Immuno-modulatory**		**Medium**	**PBMC**	**rMSC**	**Coculture**	**D3 cMSC**	**D3 cMSC-Med**	**D3 cMSC-PBMC**	**D3 cMSC-rMSC**	**D3 cMSC-Coc**
OPG		2,66	2,69	6,60	7,56	**8**,**13**	5,47	5,44	1,53	0,57
ANGPTL4		3,40	3,58	8,69	8,78	**9**,**86**	6,46	6,28	1,17	1,07
TNFRSF12A		0,51	0,66	2,81	2,99	**3**,**49**	2,99	2,83	0,68	0,51
SPON1		1,94	1,76	2,73	3,07	**4**,**98**	3,04	3,22	2,25	1,91
Gal-1		1,75	2,12	3,91	4,04	**5**,**34**	3,58	3,22	1,42	1,30
NRP2		1,74	2,34	2,79	3,45	**3**,**56**	1,82	1,22	0,77	0,11
CSTB		5,41	5,69	5,84	6,13	**6**,**48**	1,07	0,79	0,64	0,35
FAP		3,82	4,03	4,24	4,40	**4**,**66**	0,84	0,62	0,41	0,25
DKK3		4,54	4,70	5,80	6,05	**6**,**89**	2,34	2,19	1,09	0,84
A2-MRAP		2,92	3,24	3,20	3,62	**4**,**44**	1,52	1,20	1,25	0,82
RGMB		1,16	1,01	2,67	3,06	**3**,**87**	2,72	2,87	1,20	0,82
MFGE8		3,42	3,56	7,36	7,74	**8**,**80**	5,38	5,24	1,44	1,05
IL6		1,29	3,70	9,65	11,63	**11**,**89**	10,60	8,19	2,23	0,25
THY 1		2,75	3,12	6,61	7,14	**7**,**84**	5,09	4,72	1,23	0,70
N2DL-2		1,21	0,72	1,89	2,25	**2**,**49**	1,29	1,78	0,61	0,24
IDUA		-0,22	-0,41	1,43	1,77	**2**,**64**	2,86	3,04	1,20	0,86
AXL		8,52	8,64	10,01	10,16	**10**,**56**	2,04	1,92	0,55	0,39
CTSF		1,29	1,05	1,61	1,98	**2**,**04**	0,75	0,99	0,43	0,06
FAS		5,80	6,03	7,17	7,50	**7**,**99**	2,19	1,96	0,82	0,49
IL15		1,37	2,10	3,23	3,28	**3**,**79**	2,42	1,69	0,56	0,51
SKR3		1,07	1,07	1,46	1,82	**2**,**11**	1,04	1,05	0,66	0,30
Upa		4,29	4,42	9,42	9,17	**11**,**27**	6,98	6,85	1,85	2,09
LTBR		2,88	2,95	4,07	4,47	**4**,**74**	1,86	1,79	0,67	0,27
GAS6		8,72	8,98	9,09	9,51	**10**,**99**	2,27	2,02	1,90	1,48
GDNF		1,86	1,35	3,30	3,96	**4**,**95**	3,09	3,60	1,65	0,99
FS		2,83	2,51	5,72	5,75	**7**,**25**	4,41	4,73	1,53	1,50
SCF		2,41	2,39	3,07	3,27	**3**,**54**	1,13	1,15	0,47	0,27
CKAP4		2,77	2,87	4,07	4,80	**7**,**58**	4,81	4,71	3,51	2,78
PDGF-RA		1,36	1,19	1,58	1,82	**2**,**87**	1,51	1,68	1,29	1,05
Gal-3		4,24	4,49	4,82	5,01	**5**,**56**	1,32	1,07	0,74	0,55
PVR		1,75	2,07	2,14	2,43	**2**,**59**	0,85	0,52	0,45	0,16
ROBO1		2,66	2,78	3,10	3,33	**3**,**76**	1,09	0,97	0,65	0,42
PD-L1		1,41	1,51	1,80	2,01	**2**,**36**	0,95	0,85	0,56	0,35
C1QTNF1		8,18	8,37	8,63	9,95	**9**,**85**	1,67	1,48	1,22	-0,10
NMNAT1		1,69	2,26	1,87	2,66	**3**,**57**	1,88	1,31	1,70	0,91
ADA		2,51	3,12	2,95	3,07	**3**,**41**	0,90	0,29	0,46	0,34
JAM-B		0,80	1,01	0,99	1,27	**1**,**42**	0,62	0,41	0,43	0,15
FSTL3		1,90	1,89	2,02	2,10	**2**,**67**	0,77	0,78	0,65	0,57
BLVRB		2,68	3,07	2,88	3,07	**3**,**88**	1,21	0,81	1,01	0,81
EZR		0,52	0,69	0,67	1,13	**1**,**27**	0,75	0,59	0,60	0,15

The categories “Consumed by MSC”, “Conditioning”, “Inhibited”, and “Immunomodulatory” are constituted by comparisons between amounts contained in the different secretomes, expressed as NPX. All 177 proteins significantly deregulated were considered initially. The mean values of NPX are represented in left rows for each category: Medium alone, PBMC alone, rMSC alone, Coculture of MSC and PBMC, and D3-cMSC. In right rows are represented the results of the substraction, protein by protein, of each category to one reference category set in bold. (a) To establish the proteins consumed by MSC, we observed which were present in higher amounts in medium alone (in bold) while reduced by other culture conditions (rMSC, coculture, D3-cMSC) and positive values were retained after subtraction. Of note, PBMC did not use these proteins and substractions were close to zero. (b) To unveil the proteins potentially involved in conditioning, we observed which one were higher in coculture (in bold) than in the other categories, and positive values were retained. (c) To unveil proteins inhibited by the conditioning, we observed which ones were lower in D3-CMSC (in bold) than in the other categories, and negative values were retained. (d) To unveil the proteins potentially harboring immunomodulatory capacities, we observed which ones were expressed higher by the D3-cMSC (in bold) than by other culture conditions, and positive values were retained

### NSG-MG mouse model

#### Outline and thymus grafting procedure

The work has been reported in line with the ARRIVE guidelines 2.0. The functional efficacy in vivo was assessed in a humanized mouse model of MG that was previously developed and validated [34]. Animal experimentations were authorized by the French local ethical committee, following guidelines of the European Union and were performed under supervision of authorized personals in the animal facility of Sorbonne University. NOD-SCID IL-2Rγ^null^ (NSG) mice were obtained from Charles River laboratories and bred in our facilities under specific pathogen-free conditions, fed ad libitum, under a 12-hour light cycle, in an environment enriched by sizzle nests. Mice of both sex aged of 14 to 30 weeks were anesthetized (intraperitoneal injection of 80 mg/kg ketamine and 7 mg/kg xylazine) and subcutaneously engrafted in the lower backs with 3 to 4 pieces of approximately 5 mm per side of freshly collected human MG thymic biopsies following Sudres et al. protocol [34] under aseptic conditions. For each thymus, 12 to 15 mice were transplanted. To reduce pain associated to surgery, mice received Buprenorphine subcutaneously (0,05 mg/kg) 30 min before intervention, and every 12 h for 48 h thereafter. Animals were watched daily. Clinical evaluation of mice was performed once a week and blood samples were obtained fortnightly and at sacrifice for follow-up of the presence of human cells. The animals were euthanized 6 or 7 weeks after transplantation by cervical dislocation. Animals that presented a weight loss > 20% within one week, or a very hunched posture, tremors, signs of dehydratation or paralysis at any day were also sacrificed.

#### Mice humanization follow-up by flow cytometry

The percentages of human CD45^+^ cells in mice blood were assessed by flow cytometry. Blood samples were treated with BD lysing buffer (BD biosciences, RT, 10 min) to eliminate erythrocytes. Cells were washed and stained with anti-human CD45 (BD, clone HI30, 4 °C, 30 min). Viability of cells was assessed using the LIVE/DEAD™ Fixable Near-IR Dead Cell Stain Kit (Invitrogen). After washing, cells resuspended in PBS were acquired using a CytoFlex S (Beckman-Coulter) and analyzed using FlowJo software. Due to obligatory lockdown (COVID 19 crisis), the first cohort of 12 animals had to be sacrificed after the 6th evaluation. Then blood cells were frozen in a mixture of DMSO (10%) and FBS (90%) and the percentages of CD45^+^ cells were evaluated after resumption of activities.

#### Mice treatment

Based on the description of the model and the effect of cMSC previously observed and quantified [34], the number of animals was estimated around 10 in each group, using a power of 80%, an alpha risk at 5%, the effect size at 0.22 (clinical score decrease in arbitrary units) and SD at 25%. We could constitute 3 groups of 9 animals each, i.e. 27 animals received human thymus grafts, 12 from one donor and 15 from a second donor. The animals were ranked according to their percentage of circulating human CD45 + cells in blood 2 weeks after grafting (low, medium or highest content). Treatments were attributed to the animals randomly inside each group constituted after ranking. Two weeks after transplantation of MG thymic fragments, 5 × 10^5^ of rMSC or cMSC suspended in 50 µL of isotonic NaCl, or NaCl alone, were injected into mice through the intra-caudal vein using an insulin syringe equipped with a 29G needle, under laminar flow hood. This dose is in the range established previously [34] (2.10^5^–1.10^6^) and fit with the usual practice in preclinical assays in mice models [56]. The pseudonymization of the cells (rMSC, cMSC) and placebo was done after the step of cell culture by a person that was unaware of the experiment and syringes labelled accordingly. The operator who injected the mice was blinded to the treatment and did not participate to the preparation of cells, nor clinical evaluations, nor data analysis. Each cage contained animals of the different treatment groups. The cages were inserted in the same rack, in the same room of the animal facility. Behavior and weight of animals were followed, as part of the clinical follow-up, and to control the health and status of the animals.

#### Clinical evaluation and scoring for NSG-MG experiments

The MG-like clinical status was assessed weekly in a blinded fashion by an operator unaware of the treatment using a composite score including behavior observation, weight loss, grip test and inverted grid test. Mouse behavior was graded on a scale of 0 to 3: score 0, no sign; score 0.5, slight tremors, hesitations; score 1, abnormal movements (walking with head and tail down); score 1.5, low exploration, head and tail down, slow motion; score 2, reduced motility and hunched posture, poorly maintained coat; score 3, paralysis, dehydration or death [34, 57]. The weight loss was scored from 0 to 3 according to the percentage of loss in grafted animals compared to sham mice (< 5% = score 0; 5-9.99% = score 1; 10-14.99% = score 2; ≥15% and death = score 3). The muscle strength was analyzed by measuring the forelimb strength with a grip strength apparatus (GRIPTEST Bioseb, France) after a 5-minute run on a treadmill and comparing the weekly obtained values to the ones obtained during habituation (prior onset of symptoms). Muscle strength loss was scored from 0 to 4 (No loss or slight increase due to training = score 0; <10% = score 1; 10-19.99% = score 2; 20-29.99% = score 3; ≥30% and death = score 4). The elapsed time that mice resisted to fall from inverted grid (Tr) was assessed for a maximum of 60 s and scored from 0 to 3 (Tr = 60 s, score 0; Tr = 45 to 59 s score 1; Tr = 44 − 30, score 2; Tr < 30 s or death, score 3). A weekly global clinical score was calculated as follows: weight loss score + strength loss score + average obtained from (behavior score + inverted grid score). As the clinical signs reflect the status of the MG donor [34], the global clinical scores of each animal were normalized to its scores at the time of cell injection. Due to obligatory lockdown (COVID 19 crisis), the first cohort of 12 animals had to be sacrificed after the 6th evaluation. The blinding was lifted after these calculations and groups reconstituted according to treatments.

### Statistics

Differences between independent experimental groups were analyzed using GraphPad Prism 10 software. When 2 groups were compared, unpaired T-test was applied. When more than 2 groups were compared, ANOVA test for multiple comparisons was used (Tukey’s). The test is specified in the figure legend. Statistical significance was recognized at pvalue inferior to 0.05. In all figures the significance is displayed as asterisks, as follows: *P <.05, **P <.01, ***P <.001, ****P <.0001, except when indicated.

## Results

### Conditioning using PBMC triggers the expression of a specific panel of genes

**Fig. 1:**
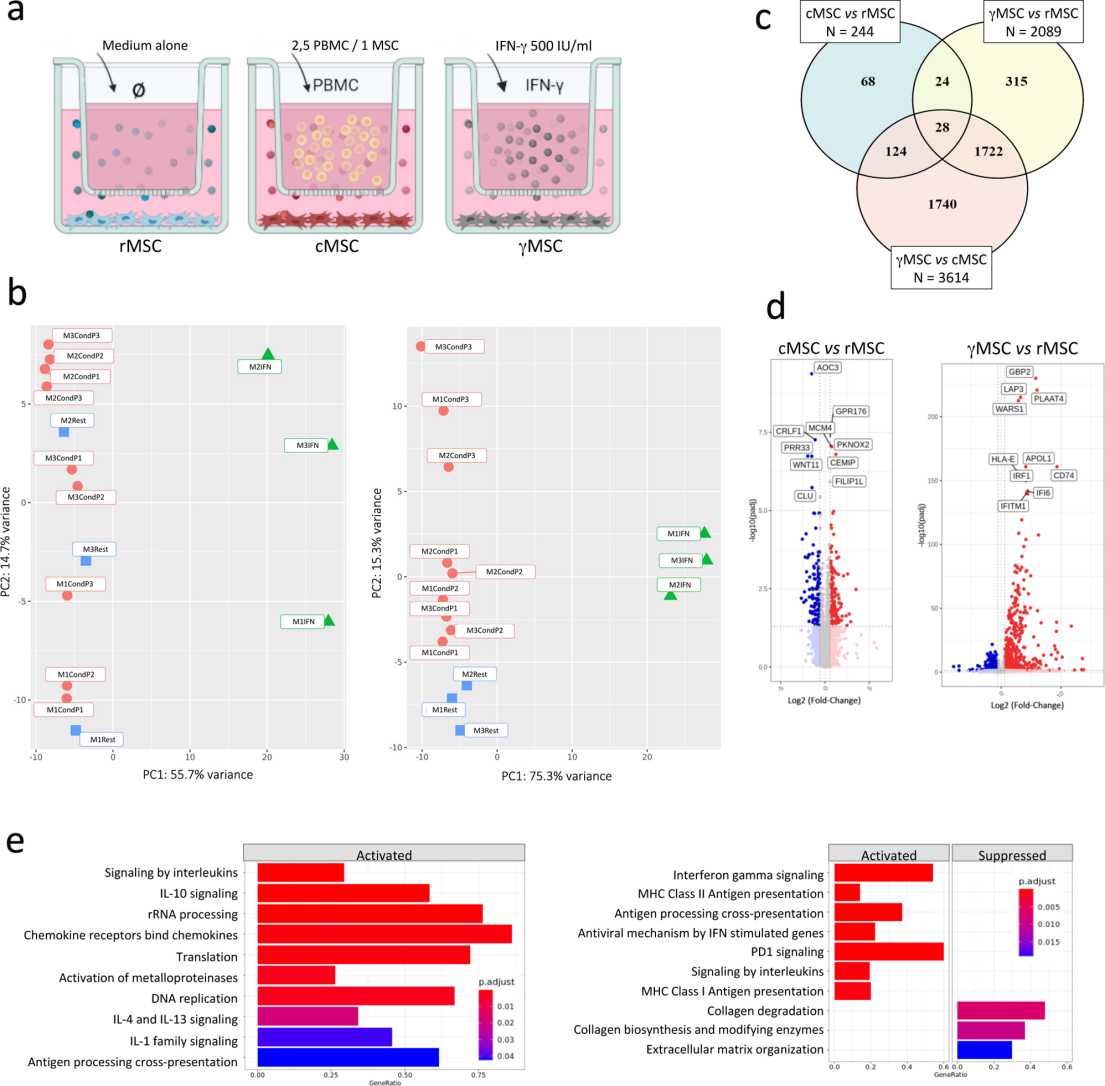
Gene signature of MSC under different treatments RNA sequencing analysis was conducted under different treatments. (**a**) Schematic representation of the different cell samples used for RNAseq study: non-stimulated MSC (rMSC, *n* = 3), MSC obtained after coculture with PBMC for 72 h (cMSC, *n* = 9 combinations) and MSC after stimulation with 500U/ml of INF-γ for 48 h (γMSC, *n* = 3 cultures). (**b**) PCA analysis of 500 most variable genes in the transcriptome before (left) and after (right) removal of batch effects from donor using the *removeBatchEffect* from the *limma* v3.50.3 package. (**c**) Venn diagram showing the number of shared and unique differentially expressed genes (DEG) found between compared conditions. The total number of DEG is shown in cases attached to each diagram. (**d**) Volcano plots visualization of RNAseq data comparing cMSC versus rMSC (left) and γMSC versus rMSC (right). The top 10 DEG with highest padj value were annotated. (**e**) Most pertinent activated (or suppressed) pathways, determined from GSEA. Each plot displays pathways in Reactome and Gene Ontology Biological process (GO_BP) collections in cMSC (left) and γMSC (right) when compared to rMSC. Bar height represents gene ratio and bar color the padj value

Primary adipose-tissue derived MSC cultures from 3 donors were left untreated, grown for 72 h in coculture with PBMC from 3 different donors, or grown for 48 h with IFN-γ as outlined in Fig. 1a. The RNAseq study compared the levels of gene expressions by MSC between these treatments and was performed by the Genom’ic Core Facility at the Institut Cochin. An unsupervised principal component analysis (PCA) was performed based on the expression of the 500 most variable genes (Fig. 1b). The first plot pointed-out the strong genes’ modulation induced by IFN-γ treatment, accounting for 75% of the dataset overall variability (Fig. 1b left), confirming its well-known potent effect on the MSC transcriptome. The plot also pinpointed a MSC donor effect, as samples were primarily grouped by MSC donor in PC2. The PCA was re-plotted after removing the donor effect (Fig. 1b right) and in this new representation, it reflects the effect induced by cellular conditioning. PBMC 1 and 2 induced the same range of effects, while PBMC 3 induced a more dramatic change, suggesting that the magnitude of conditioning is more dependent on the PBMC than on the MSC donors. The PCA projection also suggests that the priming by IFN-γ limits the heterogenity between MSC.

Heatmap of the 80 most differentially expressed genes comparing samples of each pair of conditions (rMSC vs. cMSC, γMSC vs. rMSC and cMSC vs. γMSC), underlined the homogeneity of gene expression within groups, and allowed a segregation between treatments (Supplemental Fig. 1a-c).

To understand changes in gene regulation in MSC upon conditioning with PBMC or priming by IFN-γ, we identified differentially expressed genes with an adjusted p value (padj) < 0.01. As shown in the Venn diagram (Fig. 1c), when compared to the resting state, the number of genes dysregulated by PBMC conditioning (244) was 9 times lower than the number of genes dysregulated by IFN-γ (2089). From them, 124 and 1103 genes were up-regulated, and 120 and 986 genes were down-regulated by PBMC conditioning and IFN-γ priming, respectively. Only 52 differentially expressed genes (DEG) were common to both treatments. When comparing cMSC and γMSC gene expression, the number of dysregulated genes was even higher (3614) with 1838 genes up and 1776 down, respectively. The Supplemental Table 3 presents the 45 top genes upregulated by coculture *versus* resting, by IFN-γ *versus* resting, and by IFN-γ *versus* coculture. Only 1 gene out of 45 is similarly upregulated both by coculture and IFN-γ when compared to resting. Meanwhile, 35 out of 45 genes are simultaneously upregulated by IFN-γ when compared to their level in coculture and resting. Together, these observations suggest treatment-dependent transcriptomic signatures and a molecular specificity of PBMC conditioning.

To visualize gene modulation by each treatment, volcano plots were generated with representation cut-offs set horizontally at -log10 (0.05) and vertically at log2 (1.5) (Fig. 1d). The top 10 modulated genes with highest padj value were annotated. Results revealed that the PBMC conditioning induced slight changes when compared to rMSC transcriptomic profile with log2 fold changes falling between 1 and 5 and -log10 padj < 10. Among the most significantly dysregulated genes several were involved in migration or matrix modeling (*AOC3*,* CEMIP*,* PRR33*), cytokine production (*GPR176*,* CRLF1*), proliferation (*MCM4*,* WNT11*,* PKNOX2*) and chaperone protection (*CLU*). By contrast, IFN-γ induced highly significant dysregulations with modulations reaching values > 5 for log2 fold-change and > 100 for -log10 padj. Unsurprisingly, the most dysregulated genes are involved in classical IFN-γ pathways and related to host defense (*HLA*,* CD74*,* APOL1*,* IFITM1*,* IFI6*,* GBP2*,* LAP3*,* PLAAT4*,* WARS1*,* IRF1*).

Gene Set Enrichment Analysis was performed and enriched pathways were identified using padj < 0.05 as cut-off. The most informative databases were Reactome and GO for its “Biological process” category. The 10 most pertinent pathways for each treatment and on each category were represented as bar plots (Fig. 1e**)**. In cMSC, enriched pathways included those related to an active cellular and metabolic state (DNA replication, rRNA processing and translation), to extracellular matrix (ECM) remodeling and migration (ECM disassembly, activation of matrix metalloproteases (MMP), cell chemotaxis and migration), to paracrine and autocrine communication (signaling by several different interleukins and chemokines) and to immunomodulation (regulation of defense and inflammatory responses). As expected, principal enriched pathways in γMSC included IFN-γ signaling, NF- κB signaling, inflammatory response, signaling by interleukins and those that mimic the anti-viral response. Interestingly, these cells down-regulated pathways related to ECM remodeling. Therefore, different and/or complementary mechanisms were exerted by PBMC and IFN-γ.

**Fig. 2:**
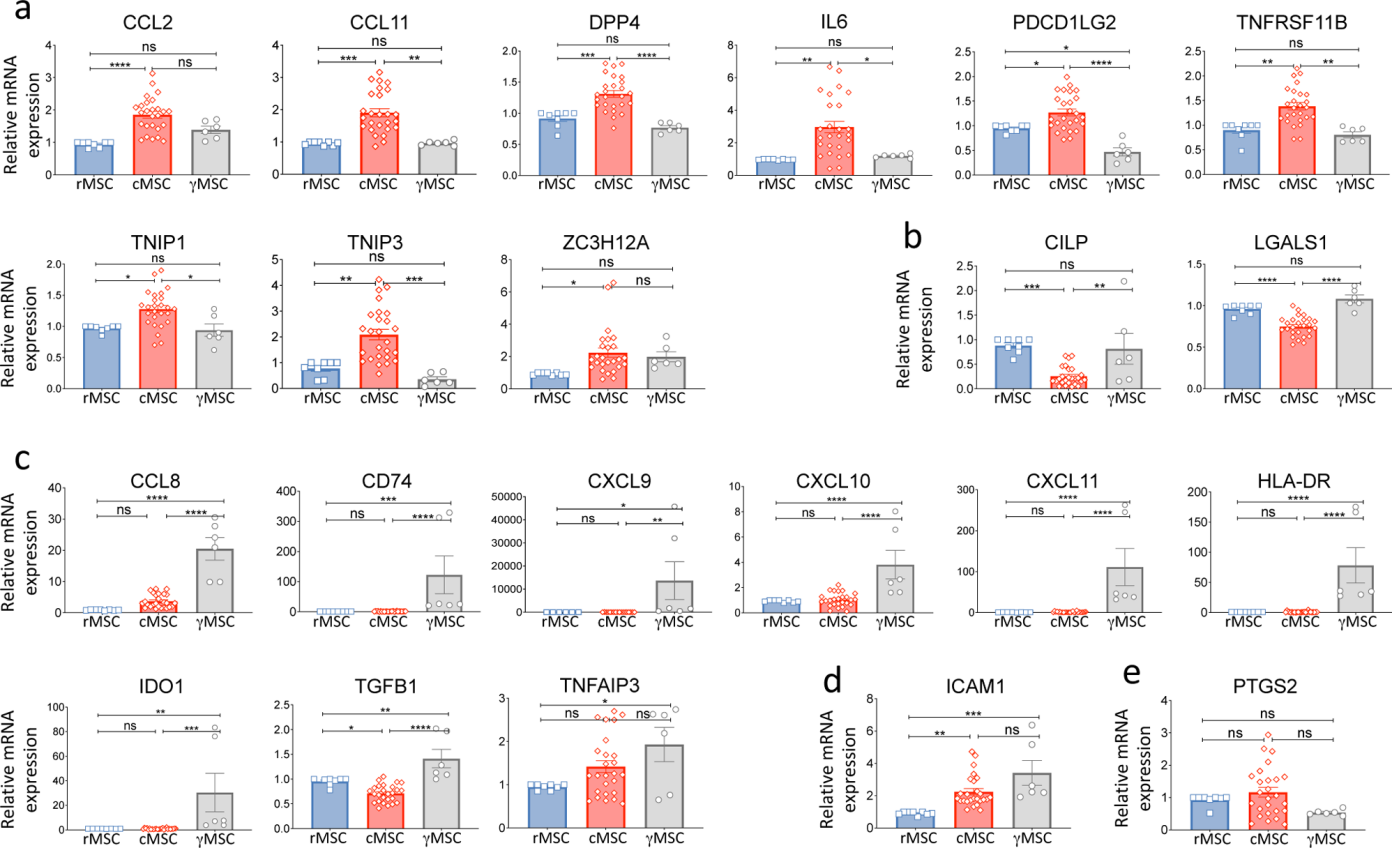
Gene expression assessment by RT-PCR. Gene expression was first normalized to the reference gene GAPDH and then presented as mRNA expression relative to rMSC of each culture. The graphs are grouped in categories, according to the expression of the given gene under the effect of treatment. (**a**): increased by PBMC conditioning; (**b**) decreased by PBMC conditioning; (**c**): increased by IFN-γ; (**d**): increased by both treatments; (**e**): no effect. Data were analyzed using One-way ANOVA and are presented as mean ± standard error of the mean. The top bar compares rMSC and γMSC, the bottom bars compare cMSC with rMSC and γMSC. *P ≤.05, **P ≤.01, ***P ≤.001, ****P ≤.0001

To validate and refine the informations brought by RNASeq study, we analyzed the expression levels of candidate genes by RT-qPCR. A short list of candidates included genes extracted from the RNASeq study. *PDCDLG2* was added to the list after the observation of its impact in immunomodulation (flow cytometry analysis, personal oservation, data not shown). Gene expression was formulated as rMSC relative mRNA expression. As expected, differential expressions were observed among conditions, displaying 5 scenarios (Fig. 2): (a) Up-regulated genes upon PBMC conditioning (*CCL2*,* CCL11*,* DPP4*,* IL6*,* PDCD1LG2*,* TNFRSF11B*,* TNIP1*,* TNIP3 and ZC3H12A*); (b) Down-regulated genes upon PBMC conditioning (*CILP*,* LGALS1*); (c) Up-regulated genes upon IFN-γ priming (*CCL8*,* CD74*,* CXCL9*,* CXCL10*,* CXCL11*,* HLA-DR*,* IDO1*,* TGFB1*, and *TNFAIP3*); (d) gene upregulated by both treatments (*ICAM1*); and finally, (e) gene with equivalent expression in all conditions (*PTGS2*).

The results of the RNASeq analysis were coherent with gene expression analysis in 19 cases out of 21 (90%). In two occasions (*IL6* and *TNFAIP3*), a discrepancy was noted between these analyses. Regarding *IL6* expression, while the level in cMSC was increased as expected, it was also expected higher in γMSC. Regarding *TNFAIP3*, the level in cMSC was higher than in γMSC as expected, but this increase was not significant. Altogether, these results underlined the differences in gene regulations between PBMC conditioning and IFN-γ priming suggesting distinct mechanisms of action.

### Differential phenotypical hallmarks of MSC conditioning by PBMC

**Fig. 3:**
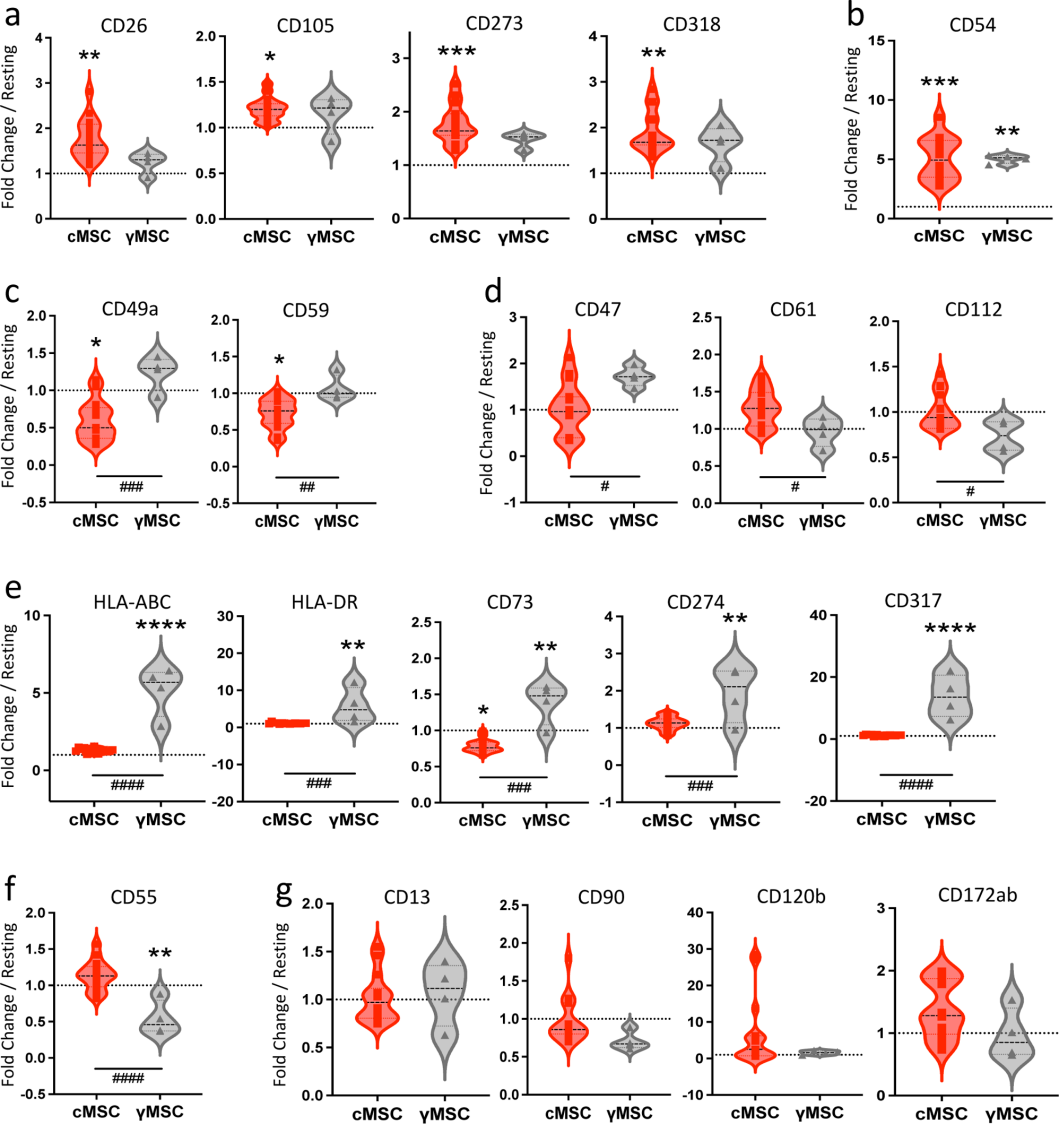
cMSC phenotypical signatures established by flow cytometry. rMSC (*n* = 4), cMSC (*n* = 15), and γMSC (*n* = 4) were analyzed for the expression of extracellular markers using flow cytometry. Differences in the expression of markers between conditions are represented as fold-changes of their respective rMSC counterparts, represented by dashed lines. (**a**) Markers up-regulated by PBMC conditioning; (**b**) Marker increased by both PBMC conditioning and IFN-γ priming. (**c**) Markers decreased by PBMC conditioning, or (**d**) differentially regulated according to treatment; (**e**) Markers up-regulated by IFN-γ priming; (**f**) Marker decreased by IFN-γ priming; (**g**) Markers showing no significant evolution, and/or high variability among experiments. Data were analyzed with One-way ANOVA test. Statistical significance between cMSC and rMSC are represented at the top of figure by stars: *P ≤.05, **P ≤.01, ***P ≤.001, ****P ≤.0001. Statistical significance between cMSC and γMSC are represented at the bottom of figure by hashtags: #*P* ≤.05, ##*P* ≤.01, ###*P* ≤.001, ####*P* ≤.0001

To establish the differential phenotypic signatures of cMSC and γMSC by flow cytometry, 23 samples were used (4 rMSC, 15 cMSC, 4 γMSC) to compare the expression of 20 markers found dysregulated in our study (CD26, CD54, CD120b, CD274, CD317, CD318, HLA-DR) or selected from the literature (CD13, CD47, CD49a, CD55, CD59, CD61, CD73, CD90, CD105, CD112, CD172ab, CD273, HLA-ABC). Figure 3 presents treatment-related differentially expressed markers when reported to the resting state and grouped by categories. Interestingly, we observed that CD26, CD105, CD273 and CD318 were increased following conditioning (Fig. 3a), and CD54 was increased by both treatments (Fig. 3b). CD49a and CD59 were decreased by conditioning (Fig. 3c). Significant differences between cMSC and γMSC were noted for CD47, CD61, CD112 (Fig. 3d). On the other hand, HLA-ABC, HLA-DR, CD73, CD274, and CD317 were increased by IFN-γ treatment (Fig. 3e), while CD55 was decreased (Fig. 3f). Important variability was reported regarding CD120b and CD172ab expressions, and no significant variation was observed regarding the expression of CD13 and CD90 by cMSC (Fig. 3g). These results were coherent with the outputs from the RNAseq dataset and with the PCR validation of CD26, CD54 and HLA-DR shown above. They provided specific markers for characterizing PBMC conditioning, such as CD26, CD273, CD318 which may be used for their validation.

### Characterization of metaclusters by mass cytometry (CyTOF)

To define specific groups of sub-populations and their changes among rMSC, cMSC or γMSC, or to validate their potential homogeneities, we conducted single-cell mass cytometry analysis of 30 cell preparations (4 rMSC, 22 cMSC, 4 γMSC). The flow cytometry experiments allowed the identification of informative markers to build-up an MSC-dedicated mass cytometry Ab panel. After testing, 24 functional metal-tagged Ab were retained, to which we added 3 Ab directed against the intracellular targets PTGS2 (prostaglandin E2 synthase), IDO-1 (indoleamine-2,3dioxygenase) which are classically involved in immunomodulation, and Ki67 (a proliferation marker) (Supplemental Table 1).

**Fig. 4:**
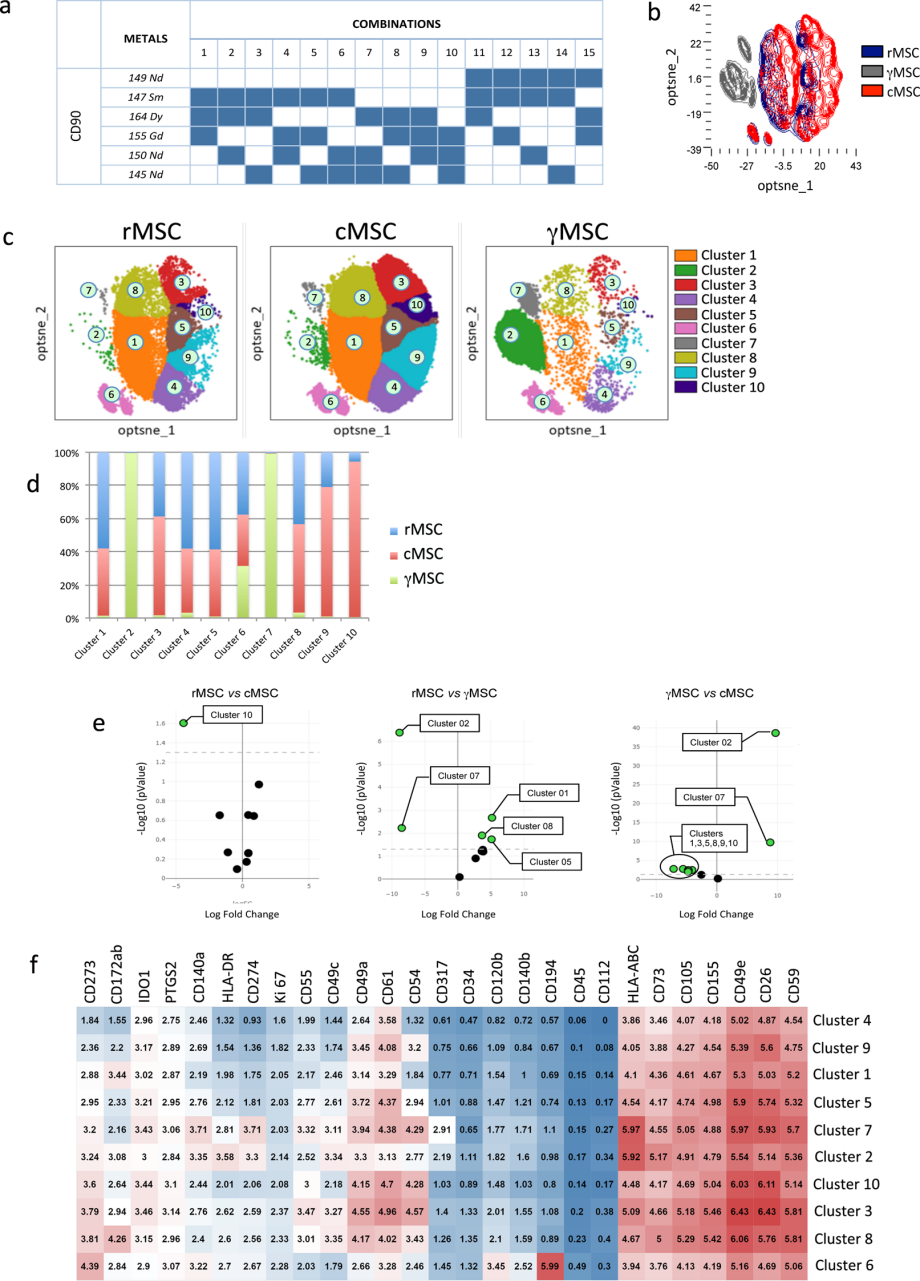
Unsupervised and supervised analysis of MSC phenotypic profile by CyTOF rMSC (*n* = 4), cMSC (*n* = 22), and γMSC (*n* = 4) were barcoded and stained with a dedicated home-made panel and analyzed by CyTOF. Data analysis was performed using Omiq software. (**a**) Table showing barcoding strategy based on CD90 staining. Each sample was identified by a unique combination of 3 different metal-tagged CD90 Ab, then samples were distributed in 2 tubes gathering 15 each. (**b**) Unsupervised optimized t-Distributed Stochastic Neighbor Embedding (Opt-SNE) plot overlaying rMSC, cMSC and γMSC samples. (**c**) Scatterplot showing metaclusters yield by each MSC condition. Ten metaclusters were identified using ClusterX analysis tool and each one is represented by a different color and identified by a number. (**d**) Histogram showing the frequency of cells in each of the identified metaclusters for each MSC condition. Most cells (> 50%) were belonging to clusters 1, 4, 5 in resting conditions. Most cells were belonging to clusters 3, 8, 9 and 10 after PBMC conditioning. The most important difference between resting and conditioning was observed in cluster 10. Following IFN-γ priming, most cells were concentrated in clusters 2 and 7. (**e**) Volcano plots presenting the significantly modulated clusters between 2 conditions: rMSC *versus* cMSC (left), rMSC *versus* γMSC (middle) and γMSC *versus* cMSC (right) (cut-off p value ≤ 0.05). (**f**) Heat-map showing the expression of markers for each metacluster, expressed in mean mass intensity units. The color intensity (red or blue) reflects the relative weight of a given value in its column, from the highest in intense red to the lowest in intense blue

Classical CyTOF barcoding requires fixation/permeabilization protocols that can alter surface antigens by introducing conformational changes and irreversibly modifying antigenic epitopes recognized by Ab, therefore surface barcoding strategy has the advantage to preserve cell phenotypic profile. Our samples were surface barcoded using an original approach based on the combination of Ab directed against CD90 tagged with specific metals (Fig. 4a), given that CD90 expression is a hallmark of MSC. To avoid steric hindrance, different clones of CD90 Ab were used. Each sample was attributed a unique labeling code composed of 3 different metal-tagged CD90 Ab.

Dimensional reduction of the 27 analyzed parameters was performed taking all samples into account and using the optimized T-distributed stochastic neighbor embedding (opt-SNE) algorithm; the resulting map is presented in Fig. 4b. In this figure, samples were contour-colored based on their condition (rMSC, cMSC and γMSC) and we observed that the overall phenotypic profile of rMSC and cMSC were close as evidenced by their highly overlapping representations. In contrast, γMSC had a completely different profile that did not superpose to any of the former ones, but was very homogenous. We then categorized single-cell data into ostensible cellular metaclusters using ClusterX algorithm. The projections defined 10 different metaclusters (Fig. 4c) which were tightly connected. While cMSC and rMSC clustering profiles were closely related, γMSC ones presented different distributions and intensities. This further confirms the differential effects of PBMC conditioning and IFN-γ priming on MSC.

For each MSC condition, the proportion of each cluster was analyzed (Fig. 4d). Upon PBMC conditioning, most metaclusters were conserved when compared to the rMSC ones, nevertheless one metacluster (number 10) was more than 15 times over-represented. Upon IFN-γ treatment, the homogeneity of MSC increased. Indeed, 75% and 17% of cells were contained in clusters 2 and 7, respectively, totalizing 92%. The Fig. 4e underlines the significances attributed to the modulation of these metaclusters: the cluster number 10 is significantly enhanced when comparing cMSC to rMSC (left). Similarly clusters number 2 and 7 are enhanced when comparing γMSC to rMSC (middle) and cMSC to γMSC (right), and most of the remaining clusters are significantly diminished. Finally, to define the identity of the clusters, a heat map displaying markers expression for each cluster is presented in Fig. 4f. As observed above, marker expression among clusters varied mostly regarding their intensity and not the presence or absence of expression, which explains the intrinsic connection between most of them. The cluster 10, which was induced after PBMC conditioning, contained cells expressing among the highest levels of CD26, CD49a, CD49e, CD54, CD61, CD155, CD273, IDO1 and PTGS2. The clusters 2 and 7 which were induced after IFN-γ priming are mainly characterized by common high expression of CD49e, CD54, CD59, CD61, CD73, CD105, CD140a, CD155, CD274, IDO1, HLA-ABC and HLA-DR. Differences between clusters 2 and 7 included higher expression of CD54, CD61 and IDO1 and lower expression of HLA-DR in cluster 7 compared to cluster 2. These results were coherent and complementary to the results obtained by flow cytometry.

### Differential characterizations of the secretomes

**Fig. 5:**
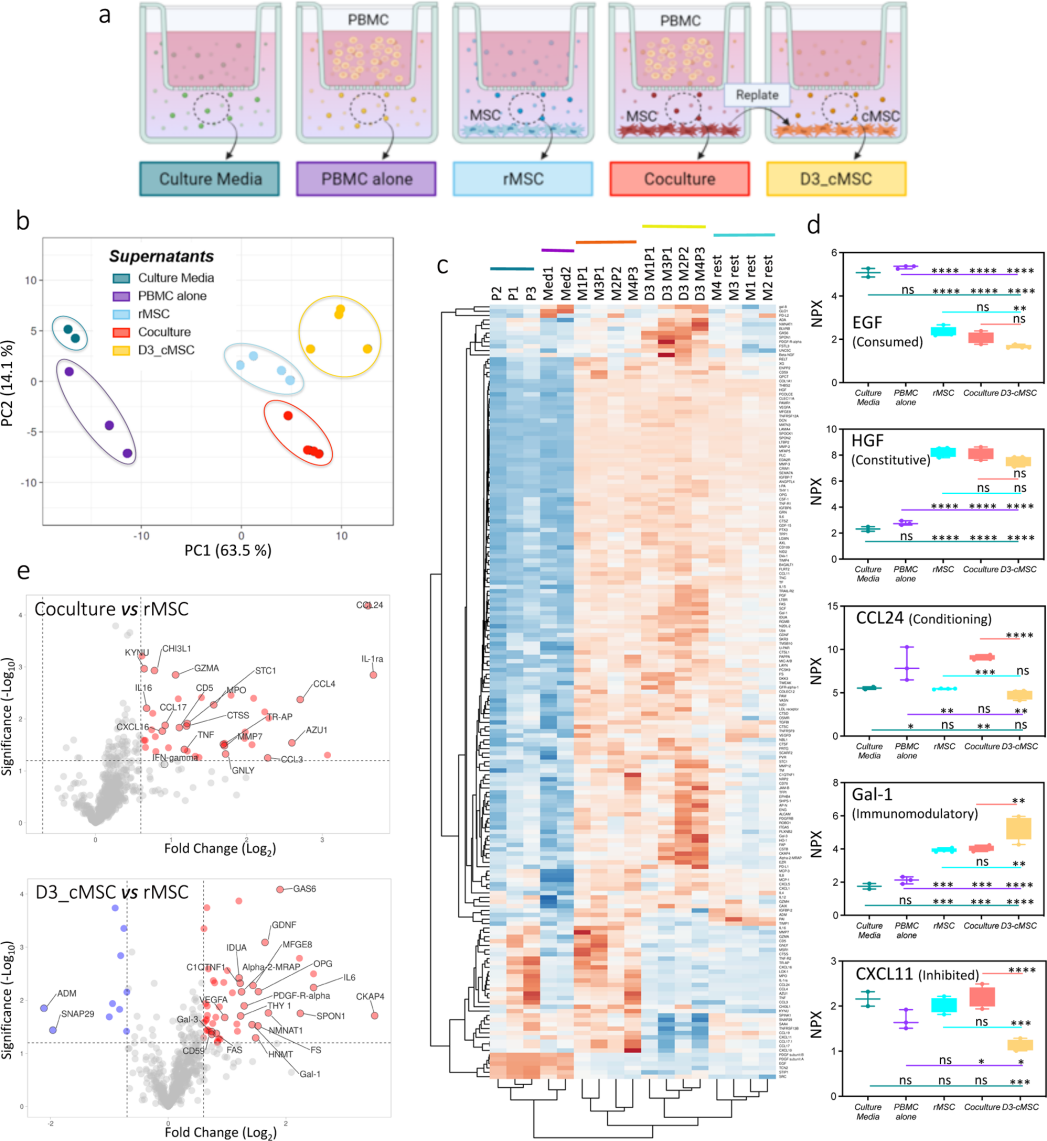
Characterization of MSC secretome Supernatants produced by MSC alone, by PBMC alone, by the culture media alone, by MSC during coculture with PBMC, and by D3-cMSC after coculture, were analyzed for the secretion of 609 different proteins using proximity extension assay methodology (Olink). Data were obtained as NPX value (Normalized Protein eXpression, Olink’s arbitrary unit expressed in Log2 scale) and explored using Olink^®^ Insights Stat Analysis app (www.olink.com). (**a**) Representation of the different samples set-ups used for supernatant preparation and the categories. Color codes were established for each sample type and used throughout this figure. (**b**) PCA plot of samples based on the differentially secreted proteins identified between at least two categories (*n* = 177). Data analysis was performed using One-way ANOVA and padj of 0.01 as cut-off. (**c**) Heat-map showing the detection of the 177 differentially secreted proteins in the different samples. P identify the PBMC samples (P1 to P3), Med identify the culture medium alone, M identify the MSC culture (M1 to M4). In cocultures the number of the MSC culture (M1 to M4) is associated to the PBMC sample (P1 to P3). The cultures harvested and replated for 3 days are identified by D3 followed by the number of the initial coculture. The columns correspond to the samples while the rows correspond to the proteins. Rows are centered and unit variance scaling is applied to them. Both rows and columns are clustered using correlation distance and average linkage, only columns dendrograms are shown for simplification. Color intensity of each grid represents the numeric differences expressed as Z-score. (**d**) Typical representations of the different groups in which the differentially secreted proteins were classified. Each group is illustrated by an example protein whose secretion profile is characteristic of the group profile, i.e. EGF plot shows the typical general secretion profile of molecules identified as “consumed by MSC”. The colored lines represent the analysis of significance between each category of sample and significances are indicated above each category. Each line color corresponds to the compared category, e.g. the green line corresponds to the comparison between medium and all other categories. The pink line corresponds to the comparison between PBMC and rMSC, coculture and D3-MSC. The blue line corresponds to the comparison between rMSC, coculture and D3-MSC. The red line corresponds to the comparison between coculture and D3-MSC. *P ≤.05, **P ≤.01, ***P ≤.001, ****P ≤.0001, ANOVA one-way test. (**e**) Volcano plot showing differentially expressed molecules upon comparison of coculture and rMSC supernatants (top), and comparison of D3-cMSC and rMSC supernatants (bottom). Representation’s cut-offs were set at log2 (1.5) (i.e. 0.6) for the fold-change and–log10 (0.05) (i.e. 1.2) for the p value. Significantly upregulated proteins are shown in red, significantly down-regulated proteins in blue and non-significantly modulated proteins in grey

To identify proteins involved in MSC conditioning or immunomodulation, we conducted a secretome analysis using the proximity extension assay developed by Olink^®^. This study exclusively focused on PBMC conditioning and we did not address here the secretome modulation induced by IFN-γ. The supernatants of cell cultures were harvested, and their contents were sent for analysis by Olink^®^ (Upsalla, Sweden) as outlined in Fig. 5a. The secretome profiles changed according to conditions, and the initial step of the analysis involved comparing the differentially secreted proteins between two or more of the 5 studied conditions (culture medium without cells, PBMC alone, rMSC, MSC-PBMC coculture, and conditioned MSC grown for 3 days after coculture and termed D3-cMSC), from the global list of 609 proteins tested. Setting padj < 0.01 as the cut-off, 177 proteins were identified. PCA analysis based on these 177 proteins (Fig. 5b) revealed relevant clustering of samples belonging to the same category. Primary observed variance was associated to the presence or absence of MSC. Indeed, medium alone and PBMC cultured alone clustered together in PC1, while differences still existed between these two groups as shown in PC2. In contrast, all MSC-involving secretomes clustered separately, with discernible differences between rMSC, coculture and D3-cMSC. To further explore the expression patterns of these 177 proteins, we generated a heatmap (Fig. 5c) and confirmed the clustering observed in the PCA, highlighting distinct expression profiles corresponding to each experimental condition. Of note, an overall homogeneity was observed within each condition, suggesting minimal variability within each group. As expected, the coculture condition did not result in the simple composition of PBMC and rMSC supernatants alone, underlining that the interaction between these cell types induced changes in the production of certain proteins. Similarly, the supernatant from D3-cMSC differed from the supernatants of rMSC and MSC in coculture, suggesting that conditioned cells produced a unique set of proteins (Fig. 5c).

Based on the profile of expression provided for each of the 177 proteins, we proposed to categorize them among 6 distinct categories (exemplified in Fig. 5d and listed in Table 1). (1) 7 proteins were highly present in culture medium and in PBMC alone but decreased in rMSC, D3-cMSC and coculture supernatants, hence they were presumably consumed by adherent cells, e.g. EGF. (2) 65 proteins were equally present in rMSC, D3-cMSC and coculture supernatants but reduced in medium and PBMC alone (data not shown); they were presumably constitutively produced by MSC, e.g. HGF. (3) 22 proteins were found more abundant in PBMC supernatants and in coculture than in medium alone, rMSC and D3-cMSC, and may be involved in the conditioning of MSC by PBMC, or in the immunomodulation by MSC, e.g. CCL24. (4) 40 proteins were found more abundant in conditioned D3-cMSC, and eventually in coculture, than in the other conditions and may be involved in the immunomodulatory capacities of cMSC, e.g. Gal-1. (5) 10 proteins were less abundant in D3-cMSC than in coculture and rMSC supernatants, and were presumably inhibited after the coculture conditioning, e.g. CXCL11. Finally, (6) 33 proteins were not classified in any of the former categories due to their particular and not patterned expression profiles (not shown).

The Volcano plot in Fig. 5e (top) dedicated to the molecules potentially involved in the conditioning of MSC by PBMC shows the increased secretion of each of the candidate proteins in coculture as compared to its level in rMSC supernatant (cut-offs were set at -log10 (0.05) and log2 (1.5)). Since the supernatants were obtained after 72 h of PBMC-MSC interaction, the category contained proteins that may promote the activation of MSC, as well as, proteins released by MSC upon conditionning. This category included AZU1, CCL3, CCL4, CCL17, CCL24, CD5, CHI3L1, CTSS, CXCL16, GNLY, GZMA, IL-1RA, IL-16, KYNU, LOX-1, MSR1MMP7, MPO, STC1, TNF, TNF-R2, TR-AP. Of note, IFN-γ was located just below the cut-off. The volcano plot in Fig. 5e (bottom) pinpointed some of the differentially secreted molecules when comparing D3-cMSC to rMSC supernatants. The up-regulated proteins (in red) were categorized as potential immunomodulatory molecules and they included CD59, FAS, FS, Gal-1, Gal-3, GAS6, GDNF, HNMT, IDUA, IL-6, Thy-1, OPG. The figure also highlighted down-regulated proteins (in blue) such as CCL11, ADM and SNAP29 that may be inhibited after coculture.

Most proteins unveiled by the secretome analysis were pinpointed by the RNASeq analysis of MSC compartment. Indeed 65 out of 65 among the proteins constitutively expressed by MSC, and 40 out of the 40 proteins ranked as imunomodulatory were identified by the RNASeq study. Among the activating ones, 12 out of 22 were not observed in RNASeq analysis: *AZU1*,* CCL3*,* CCL4*,* CCL17*,* CCL24*,* CD5*,* GNLY*,* GZMA*,* KYNU*,* MMP7*,* MPO* and *TNF*. These molecules, however, are classically produced by PBMC, and the RNASeq study searched only the genes expressed by the MSC compartments.

### Phenotypical changes of PBMC populations during the conditioning

**Fig. 6:**
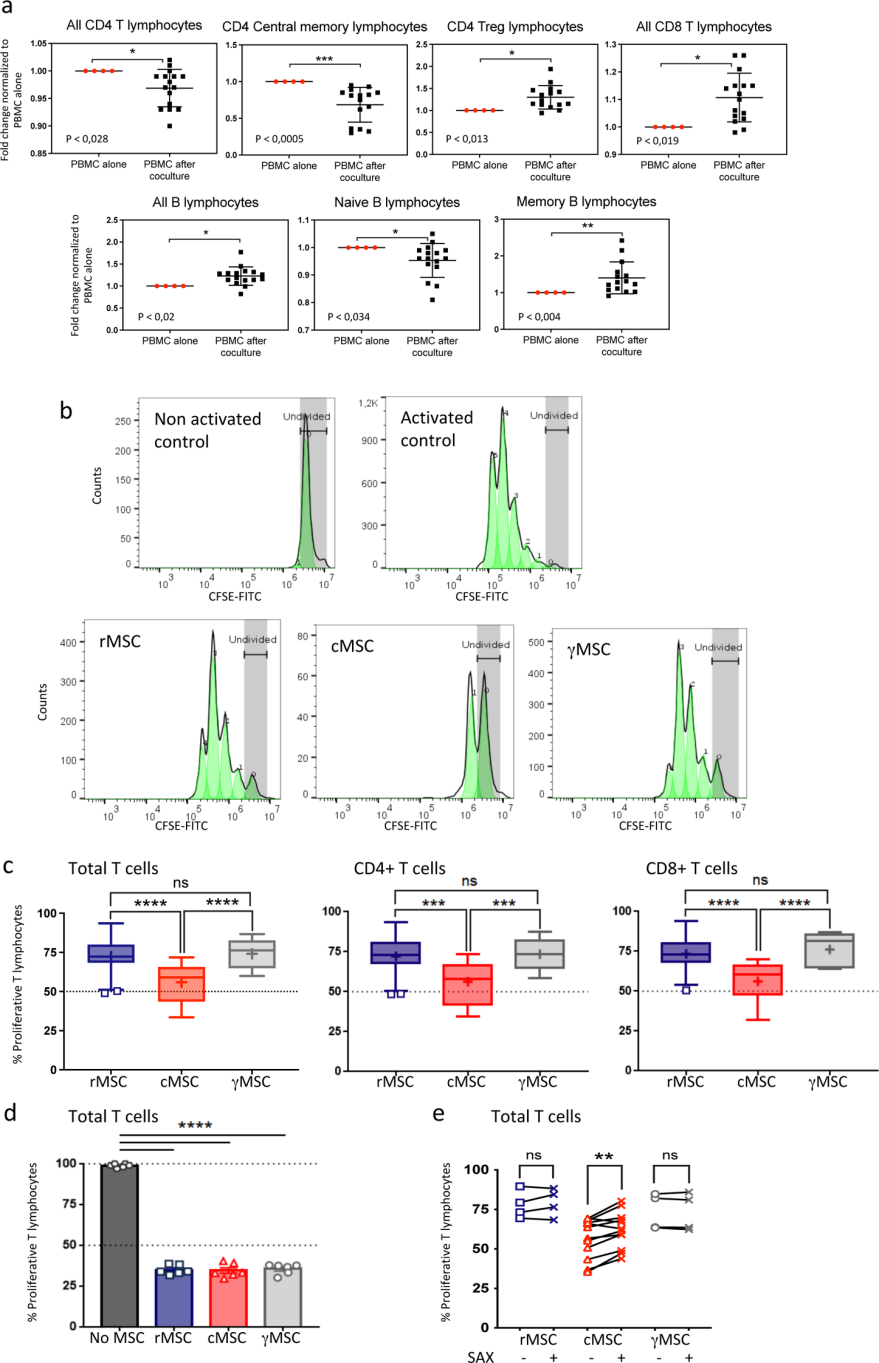
Functional assessments in vitro: changes in PBMC subsets during conditioning and capacity to inhibit proliferation. (**a**) PBMC used for MSC conditioning (*n* = 15 to 16) were harvested after the coculture step and analyzed by CyTOF using the Maxpar Direct Immune Profiling Assay™. PBMC samples cultured in growth medium alone were used as controls (*n* = 4). Changes in PBMC cell subsets after coculture are shown as fold-change relative to control PBMC. Statistical significance between conditions (Mann-Whitney test) is represented by stars: *P ≤.05, **P ≤.01, ***P ≤.001. (**b**) Set-up of the T cell inhibition assay. PBMC were incubated with CFSE and stimulated with microbeads-coupled anti CD3^+^/CD28^+^ Ab. The inhibition of T cell proliferation was assessed by flow cytometry. The histograms show representative proliferation profiles of non-activated PBMC (top left) and PBMC activated by CD3^+^/CD28^+^ coated beads (top right), and the profiles obtained in contact with supernatants produced by rMSC (bottom left), D3-cMSC (bottom middle), and γMSC (bottom right) respectively. Each green pic represents a daughter cell generation. (**c**) Inhibition of the proliferation of total T cells (left), CD4^+^ T cells (middle) and CD8^+^ T cells (right) after culture with supernatants from rMSC, D3-cMSC and γMSC. (**d**) Proliferation of T cells in absence of, or in direct contact with, MSC from the different conditions. The 50% inhibition is indicated by a discontinued line. (**e**) Proliferation of T cells when incubated with MSC supernatants from different conditions in presence or absence of saxagliptin (SAX), an inhibitor of CD26/DDP4. Data were analyzed using One-way ANOVA. ***P ≤.001, ****P ≤.0001

During coculture, paracrine interactions between both cell types are bidirectional through the Transwell membrane, then not only PBMC impact on MSC status but MSC also act on PBMC. To address the functional changes induced in the PBMC populations during the conditioning step, we used the Maxpar Direct Immune Profiling Assay™ (Fluidigm^®^), a ready-to-use CyTOF panel, to profile 37 immune cell subsets including T cells (Th1, Th2, Th17, Treg, CD4 and CD8 subsets of naïve, activated, central or effector memory), B cells (total, naïve, memory, plasmablasts), monocytes (classical, non-classical, transitional), NK cells (total, early, late), dendritic cells (plasmacytoid, myeloid), basophils and neutrophils. Modifications in cell subsets were expressed as fold-changes to their respective PBMC donor that had not been cocultured with MSC but had been grown in culture medium for 72 h using a Transwell system. We observed (Fig. 6a) that the co-culture decreased the proportion of CD4^+^ (CD3^+^CD4^+^) and CD4 central memory (CD4^+^CCR7^+^CD27^+^) but significantly increased the populations of CD4 Treg (CD4^+^CD25^+^CD127^Low^CCR4^+^) and of CD8^+^ (CD3^+^CD8^+^) cells. The proportions of B cells (CD19^+^CD20^+^HLA-DR^+^) and memory B lymphocytes (CD27^+^) were increased, while naïve B lymphocytes (CD27^−^) were slightly decreased. The proportions of monocytes (HLA-DR^+^CD11c^+^) and dendritic cells (HLA-DR^+^CD123^+^ and HLA-DR^+^CD11c^+^CD38^+^) were unchanged (not shown). These changes in proportions and activation of sub-populations provide both mechanistic clues of immunomodulation and readouts for functional efficacy of the conditioning step.

### Inhibition of T cell proliferation by MSC

**Fig. 7:**
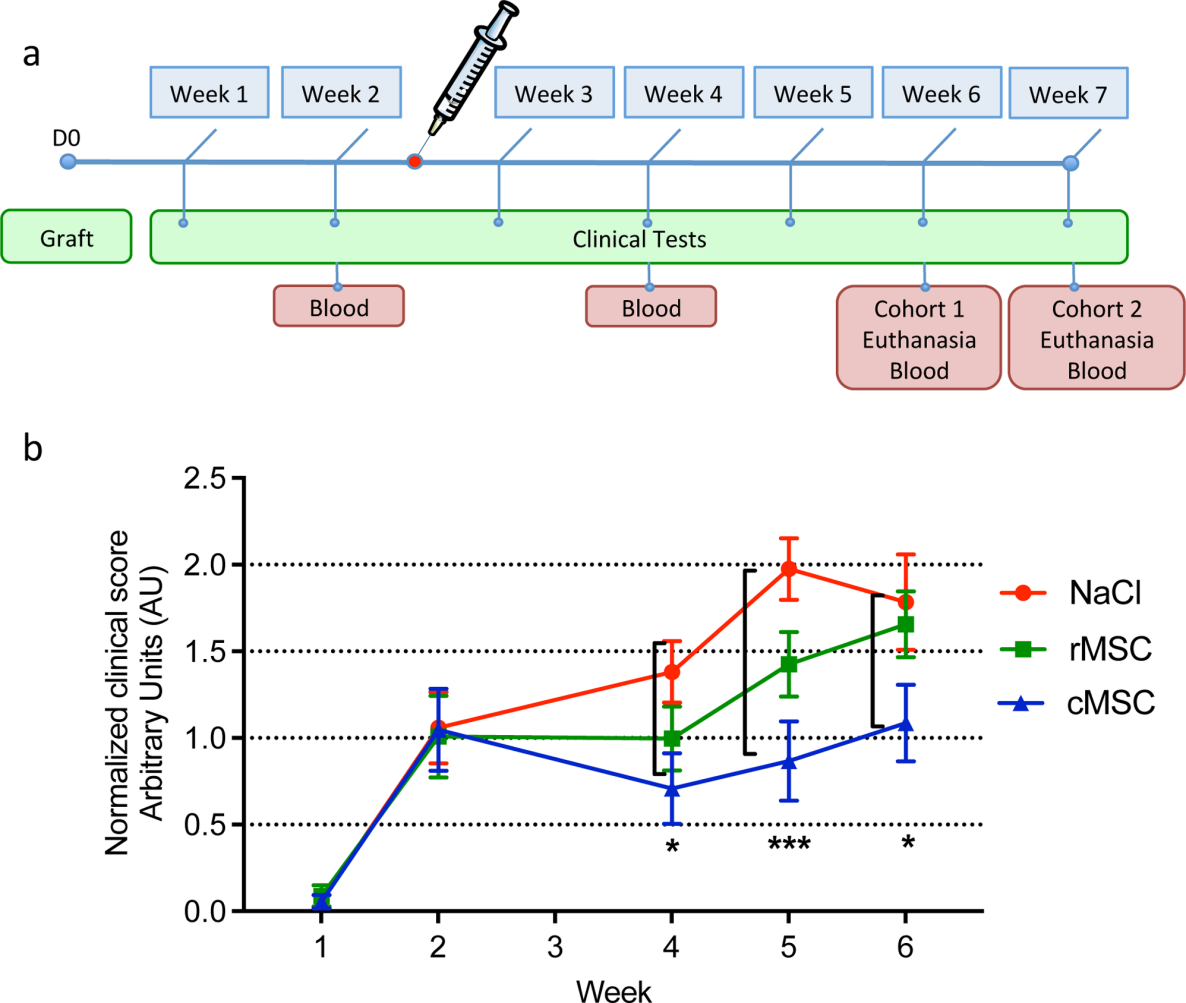
cMSC treatment effect in the NSG-MG mouse model. (**a**) Timeline of the treatment of the animal model indicating frequency of tests, and treatment of mice relative to thymus fragment grafting. rMSC or cMSC cells (5.10^5^) or vehicle (NaCl) were injected in NSG-MG mouse model in a blinded fashion (*n* = 27; 9 animals per treatment group) and the effect of treatment was tracked through composite clinical score assessment calculated based on weight loss, grip test, inverted grid test and behavior observation. (**b**) Integration of the weekly general clinical score (GCS) evolution. For each mouse the weekly registered GCS was normalized to its initial GCS (obtained at the onset of the disease, week 2). One mouse in the placebo group did not present humanization and was removed, therefore the analysis was done using *n* = 8 for this group. Normality of distribution in each treatment group was assessed (D’Agostino & Pearson test). Significant differences were observed between vehicle and cMSC–injected animals at 4, 5 and 6 weeks. Data were analyzed using two-way ANOVA multiple comparisons (Tukey’s test) and are presented as mean ± standard error of the mean. *P ≤.05, ***P ≤.001

Next, we challenged and compared the functional efficacy of rMSC, cMSC, γMSC and of their secretomes to block the proliferation of T cells activated by anti CD3/CD28 microbeads-coupled Ab, using the CFSE assay. After 72 h of activation, T cells divided prolifically and up to 5 generations of daughter cells were observed while in absence of activation cells did not proliferate (Fig. 6b). When activated T cells were placed in supernatants produced for 3 days by rMSC (*n* = 4), D3-cMSC (*n* = 12), γMSC (*n* = 3), the proportion of proliferating T cells and the number of daughter cell generations were reduced. However, the inhibition of proliferation induced by D3-cMSC supernatant was the most important (Fig. 6c**)**. Same scenario was observed when gating separately CD4^+^ and CD8^+^ T cells, both cell subsets were similarly sensitive to the inhibition (Fig. 6c). When activated T cells were grown in direct cell-cell contact with rMSC, cMSC and γMSC, the inhibition of proliferation was equivalent in the three groups (Fig. 6d). Therefore, while MSC in direct contact inhibited the proliferation of activated T cells whatever their treatment, the supernatant produced by D3-cMSC was the most efficient one. As one of the differentiating signatures of cMSC was the expression of CD26, we assessed the potential role of this molecule in the cMSC inhibitory capacity by adding saxagliptin, an inhibitor of the enzymatic activity of CD26, to the supernatant. We observed a partial but significant reversion of the cMSC supernatant inhibitory effect in the presence of saxagliptin (Fig. 7e). No significant reversions were reported for rMSC and γMSC supernatants in presence of saxagliptin.

### Modulation of clinical status in the NSG-MG mouse model

The functional efficacy in vivo was assessed as described in the outline (Fig. 7a). Twenty-seven NSG mice were grafted with fragments of thymic tissue obtained from MG patients and their clinical status was followed weekly. Twenty-six of the 27 mice presented circulating human blood cells (Supplemental Table 4) and variations were noted between animals, and depending on the thymus origin, as previously observed [34]. One mouse of second cohort presented less than 0,4% of human CD45 + cells at sacrifice, which was considered below the positivity threshold, and the animal was removed from the final calculations of effects because it was considered as a failure of humanization for unidentified reason. The rMSC, cMSC cells or placebo were administered into groups with similar levels of humanization, the 27 animals were treated (3 groups of 9 mice). The MG-like symptoms were assessed using a composite score based on 4 complementary tests (weight monitoring, grip test, clinical observation and inverted grid test). Due to obligatory lockdown (COVID 19 crisis), the first cohort of 12 animals had to be sacrificed after the 6th evaluation. Because of a grip test machine failure, the strength of mice at the third clinical assay could not be measured in the second cohort. Therefore, the results of the clinical assays at 3rd and 7th week were not included in the statistical analysis. Three mice in the placebo-treated group, 1 in the cMSC-treated group and none in the rMSC group died or reached limit endpoints and required sacrifice before the completion of the study. Animals in the first cohort presented higher contents of human CD45^+^ cells and heavier clinical signs, and the global clinical scores were normalized to the status at the time of cell injection to take into account the status of the thymus donor [34]. We observed that cMSC improved the clinical status of mice, as compared to placebo group and they reached statistical significance on weeks 4, 5 and 6 (Fig. 7b). This confirmed the observations obtained previously using MSC of research grade grown in FBS [34]. The group treated with rMSC did not show significant improvement.

## Discussion

### Context of the study

Heterogeneities of MSC is a major critical barrier to their clinical application [58, 59]. They may lie in the different anatomical origins of the samples (bone marrow, adipose tissue, cord blood, placenta, or else), in the gender, age, health status of the donors, in the method and media used for preparing the cells, in the nature of the substrates and of the culture system, in the duration of cultures and expansion levels, in the oxygen tension. The consequences of these variabilities, added to the clinical variabilities presented by the human recipients and the context of transplantation (syngeneic or allogeneic) have been detrimental, but their progressive recognition helped proposing new sets of clinical trials [20, 60, 61]. The intrinsic heterogeneity of PBMC and batch-to-batch variations are also known for long time. Each individual donor harbors a personal profile of populations, within limits, which may change with age, gender, health status or body mass index [62]. PBMC heterogeneity may even be greater than that of MSC, as revealed by potency assays combining these two cell types [30]. In this study, some heterogeneities were revealed by analysis of the PCA projection (Fig. 1). While the 3 MSC cultures evaluated in resting conditions were grouped and homogenous, they showed more heterogenity upon conditioning by the different sets of PBMC, and the donor P3 was introducing the most important heterogenity. While the age range of MSC donors was moderate, the range was wider for PBMC (20–64 years), but the fine health condition of MSC and PBMC donors was unknown for confidentiality reasons. These observations had two consequences on the study design. First, it is useful to correlate MSC surface and/or protein markers with their bioactivity in vitro and in vivo, in an attempt to identify predictive markers of potency. This settled the gene expression studies and the characterizations of phenotypic markers. Second, in a clinical perspective, this led us to consider the future replacement of PBMC by the secretome that they produce during their interaction with MSC, and this mandated the identification of the secretomes using the Olink methodology.

Several combinations of molecules are being increasingly proposed in the litterature to increase the immunomodulation capacities of MSC, including for example IFN-γ, TNF-α, IL-1β, IL-17 A, LPS, TGF-β, to offer a broader priming [21, 32, 56]. Each of these proteins exert several, complex and frequently distinct mechanisms of action, which sometimes may synergize or antagonize. To avoid these juxtapositions of effects and mechanisms, and to avoid a potential source of variability between experiments and MSC donors, we have chosen to use IFN-γ alone, whose mechanisms are described, and which has been the most used molecule for this application, as the only internal positive control.

### The secretome analysis unveils the interactions between MSC and PBMC

In this study, MSC were grown in coculture with PBMC for 72 h, which enabled prolonged bidirectional exchanges between both cell types in paracrine fashion. This 3-day long culture may mask some components with short half-life such as interleukins or prostaglandins, but the comparison of secretomes suggested significant and specific modifications of the media and allowed us to rank the identified proteins in different categories.

Some proteins, predominantly growth factors (EGF, PDGF subunits A and B), were observed to decrease in the presence of MSC, suggesting their utilization by these cells while they remained unaltered in presence of PBMC alone and in medium. Conversely, an important proportion of proteins known to be constitutively produced by MSC were observed in all cultures containing these cells. These included collagen (Col1A1), metalloproteinases (MMP3), growth factors (HGF, PGF, VEGF, GDF-15), laminins (LAMA4), receptors (TNF-R1, LDL receptor, TNFRSF9), membrane markers (CD59, CD70, CD109) and others.

Some proteins uniquely detected in the medium obtained from the PBMC-MSC coculture may be conditioning agents and some may be immunomodulatory, and both cell types may participate to their production. Because of their known pro-inflammatory function, AZU1, CCL3, CCL4, CCL24, GNLY, GZMA, IL-16, KYNU, MPO, and TNFα, may have contributed to create a pro-inflammatory environment leading to the conditioning of MSC [63–69]. These proteins are usually produced by PBMC, therefore their expression at the RNA level was not observed as the RNASeq study was dedicated to the MSC compartment only. On the other hand, CHI3L1, CTSS, CXCL16, IL1-RA, LOX1, MMP7, MSR1, STC1, TNF-R2, TR-AP may have participated to the conditioning of MSC, or to their immunomodulatory functions [70–79], and their expression was observed at RNA level.

Proteins which may play a role in MSC immunomodulatory capacity were also detected in the medium produced by D3-cMSC after being trypsinized post-coculture and replated for 72 h. These molecules included Gal-1, Gal-3, PD-L1, GAS6, IL-6, Fas, which are well reported in literature for their involvement in inflammation regulation, cell adhesion and migration and apoptosis inhibition in diverse cell types including MSC [70–85], and whose expression was also observed at RNA level. Besides, our study provides new candidate proteins potentially involved in immunomodulatory functions of MSC, which will deserve future individual investigations. Additionally, some proteins usually described as stimulators of pro-inflammatory cytokines release or highly involved in the response to IFN-γ, were down-regulated upon conditioning such as ADM, CXCL11, CCL17, CCL19 and SNAP29, consequently their down-regulation may participate indirectly to imunomodulation [86–87].

Interestingly, we detected the presence of molecules classically associated to membranes (mainly CD, e.g. CD59, CD70, CD90, CD109, CD274) whose physiological significances in a soluble form are not always documented. We should note finally that this proteomic study cannot discriminate the pure role played by extracellular vesicles.

### PBMC conditioning defines a gene expression signature

The RNAseq study suggested that several genes involved in immunomodulation were regulated by PBMC conditioning, and RT-qPCR analysis confirmed that *DPP4*,* PDCD1LG2*,* TNFRSF11B*,* TNIP1*,* TNIP3*,* CCL2*,* IL6 and ZC3H12A* genes were upregulated specifically. These genes are involved in cleavage of several interleukins and growth factors involved in immune pathways (including the CCL and the CXCL family, IGF, TNF) thus converting them in inactive or antagonistic molecules acting in a negative feedback fashion [88–90] and leading to inhibition of T cell proliferation (*DPP4 i.e. CD26*). In functional assessments, we indeed observed that inhibition of CD26 by saxagliptin partially bocked its inhibition of proliferation (Fig. 6). Some genes are involved in checkpoint inhibition (*PDCD1LG2 i.e. CD273*), inhibition of NF-κΒ pathway (*TNIP1*,* TNIP3*,* ZC3H12A i.e. MCPIP1*) [91–96], and some present pleiotropic activities, among which immunomodulation (*ZC3H12A*,* CCL2* and *IL6*) [48, 97, 98]. CD26 may also act indirectly through the induction of IL-10 [99]. Recently, it was reported that the overexpression of CD26 was a candidate marker of senescent cells, from passage 15 and beyond [100], however we used MSC cultures from passages 2 to 5 and no marker of senescence was pinpointed by RNASeq analysis. PBMC conditioning also modified the expression of genes involved in matrix modeling (*AOC3*, *PRR33*, *TNFRSF11B*), migration (*AOC3*, *CEMIP*), cytokine production (*GPR176*, *CRLF1*), proliferation (*MCM4*, *WNT11*, *PKNOX2*), and chaperone protection (*CLU*). Interestingly, a few genes upregulated by PBMC conditioning overlapped with those upregulated by IFN-γ such as *ICAM-1* which is a cell adhesion molecule playing an important role in cell-cell contact and involved in immunomodulation [101–103]. The use of IFN-γ has been widely proposed to prime human cell therapy products. However, IFN-γ usually activates several hundreds of genes involved in cellular defense against pathogens (virus, bacteria, parasites) and which are likely useless regarding immunomodulation. It triggers notably the strong expression of HLA class II molecules which participate in the immunogenicity of cells [36, 69] and facilitate their clearance. IFN-γ is involved in the activation of several pathways through expression of CD74, CD274, CXCL9, CXCL10, CXCL11, Galectins, HGF, HLA-DR, ICAM1, IDO1, IL6, MCP1, PDL1, PTGS2, VCAM [27, 31, 36, 104]. These classical modulations were confirmed in the present study, except for PTGS2 whose gene and protein expression were not upregulated in our experimental systems, but which may be influenced by variations in the primary cultures [31]. Together, our results suggest that PBMC conditioning and IFN-γ use different pathways, and that some partially overlap, in coherence with the pathway enrichment analysis results.

### PBMC conditioning defines a phenotypic signature

At protein level, the flow cytometry phenotypic analysis confirmed increased expressions of CD26, CD120b, CD318, CD273 under conditioning by PBMC, and confirmed CD54 increase by both PBMC conditioning and IFN-γ priming. The expression of integrins, intercellular adhesion molecules (ICAM-1/CD54), and other glycoproteins (CD318/CDCP1) on their surface, enable MSC to bind to T lymphocytes with high affinity [73, 103, 105, 106], then higher expression of these molecules could increase MSC-T lymphocytes interaction and immunosuppressive capacity of MSC over their targets. Elevated CD273 expression after MSC conditioning could also enhance their immunosuppressive capacities through interaction with PD-1 [105]. The phenotypic study confirmed the specific activation of some proteins by IFN-γ (HLA-ABC, HLA-DR, CD317, CD274), as expected [4, 30, 31]. Notably, CD55 and CD59, which regulate the complement pathways, were modulated by PBMC conditioning and IFN-γ in opposite directions: IFN-γ decreased CD55 but maintained CD59, whereas PBMC conditioning conserved CD55 but decreased CD59, suggesting different regulatory pathways for complement action between the two conditioning methods.

CyTOF analysis confirmed and extended the flow cytometry phenotyping. The design of our original barcoding based on the labeling of CD90 by combinations of Ab limited technical variabilities and batch effects. The CyTOF analysis documented the overall homogeneity of MSC grown in culture using hPL, since few metaclusters were observed. We did not observe the reported multilineage heterogeneity of MSC which may be due to culture conditions or the differentiation status [107]. IFN-γ treatment homogenized even more the MSC phenotypes, since most of the cells were gathered in only two metaclusters (number 2 and 7), a property of IFN-γ priming previously reported [108]. The comparative analysis of metacluster contents suggested that the signature metacluster of cMSC highly included CD26, CD49a, CD49e, CD54, CD61, CD155, CD273, IDO1 and PTGS2. The γMSC signature harbored CD73, CD105, CD140a, CD274, IDO1, HLA-ABC and HLA-DR. Interestingly, IDO1 was found significantly increased at the RNA level only in γMSC, while it was observed increased at the protein level in both γMSC and cMSC characteristic clusters. This difference underlines the complementarity of RNAseq and phenotypic analyses, as protein production and stability may not always correlate with changes in gene expression. Importantly, HLA markers were increased by IFN-γ treatment at both protein and RNA levels, but not by PBMC conditioning suggesting that the immunogenicity of cMSC would not be increased by PBMC conditioning.

Finally, both phenotypic analyses offered new combinations of markers as signatures for the validation of resting, PBMC-conditioned or IFN-γ-primed MSC. These markers will be mandated to follow the MSC status throughout their production for clinical application [19, 48].

### MSC conditioned by PBMC exert functional efficacy in vitro

Molecular and phenotypical markers are useful for quality control or to decipher mechanisms of action. However, functional markers are mandated to reflect the biopotency of the cells [18, 30] and were explored in this study. We hypothesized that the bidirectional interactions between MSC and immune cells may change the nature or proportion of immune cells in vitro, therefore we followed their evolutions by a CyTOF analysis. We observed that the proportions of CD8^+^ T cells, CD4^+^ Treg, total and memory B cells increased upon coculture, while total and central memory CD4^+^ T lymphocytes and naïve B lymphocytes slightly decreased. This is an evidence of the simultaneous involvement of several populations of immune cells in the multimodal effect of MSC. This result confirms the validity of assessing population changes as functional readouts of the conditioning step, and may become an element of a “functionality card” to be paralleled to the “phenotypic card” of cMSC.

The functional immunomodulatory capacities of MSC were also assessed in vitro through the inhibition of T cell proliferation. Direct contact between MSC and activated PBMC was sufficient to trigger inhibition, irrespective of the conditioning type. This is generally considered as a hallmark of immunomodulation capacity [17, 109]. Interestingly, supernatants conditioned by cMSC were able to produce stronger inhibitions than supernatants conditioned by γMSC and rMSC. CD26, identified in cMSC supernatant by Olink analysis, may act in soluble form and its enzymatic function can be inhibited by saxagliptin. We observed that conditioned medium produced by cMSC, when incubated with saxagliptin, loose a small but significant part of its inhibitory effect. This reversion is not observed with conditioned medium produced by rMSC or γMSC. This observation strengthens the hypothesis that CD26 is involved in the immunomodulating pathways deployed by cMSC, even if it is not solely responsible for this inhibition.

**Fig. 8:**
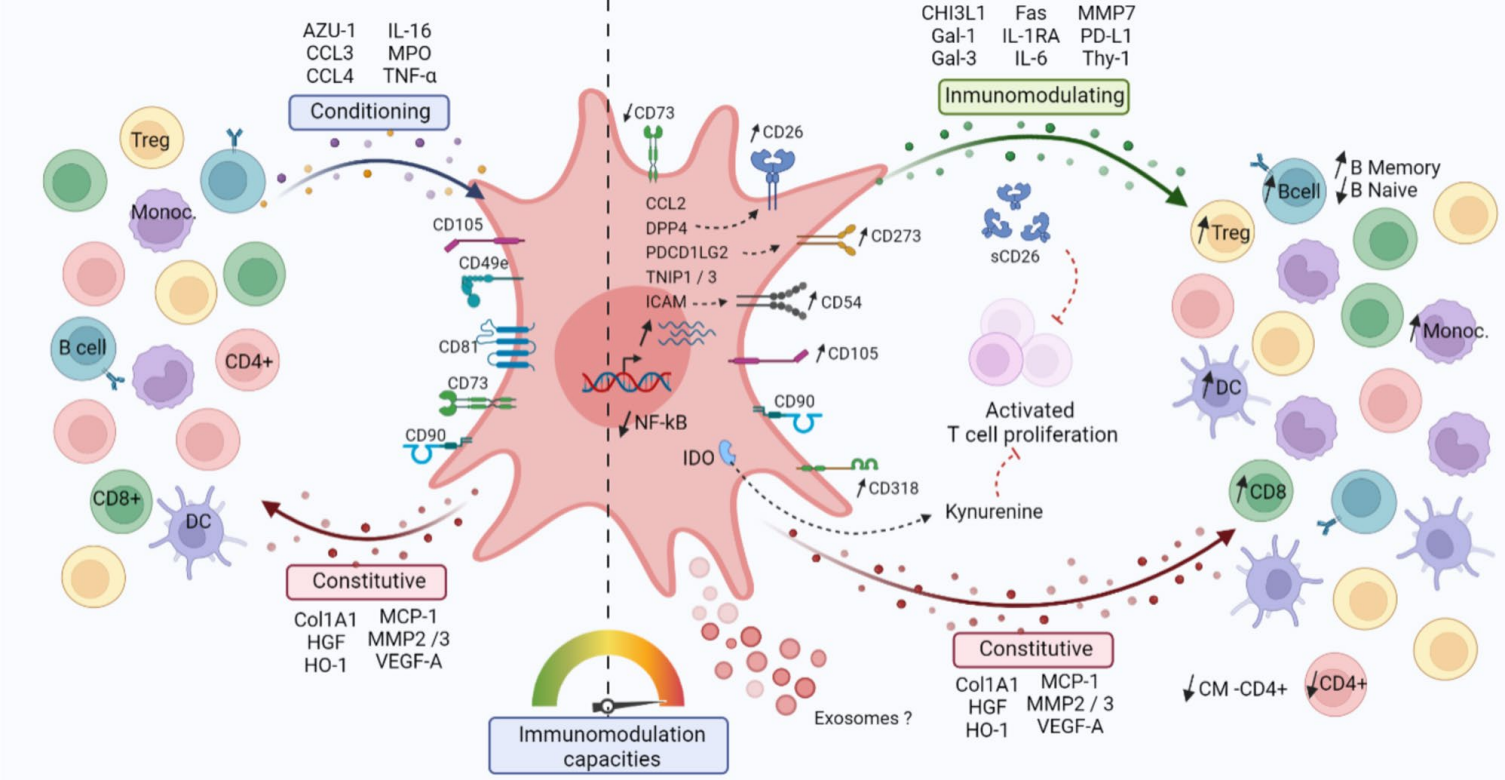
Integrated view of conditioning: induction by PBMC-MSC coculture, proposed mechanisms and immunomodulatory effects. From left to right are presented the proposed effectors, and the targets of the effects. In the center, MSC in culture continuously produce their own constitutive products (exemplified here as Col1A1, MCP-1…), expanding on both left and right sides of the figure. The interaction between MSC and PBMC produce some potentially conditioning molecules (exemplified here as AZU-1, IL-16, CCL3, CCL4, MPO, TNFα…) which act on the MSC (left side). This triggers changes in the expression of some MSC genes involved in specific pathways (NF-kB…), the expression of proteins at the membrane (CD26, CD54, CD273, CD318) or of secreted proteins proposed to participate to immunomodulation (right side, exemplified here as CHI3L1, Gal-1, Gal-3, Fas, Il-1RA, IL-6, MMP7, PD-L1, Thy-1, secreted form of CD26, IDO-1). Conditioned MSC also produce extracellular vesicles whose role was not investigated in this study. These molecules target the cells of innate and adaptive immunity, on the right part of the Figure, with various impacts depending on the nature of cells (exemplified by the overall reduction of T cell proliferation but the increase in Treg populations, the increase in B memory cells, and potential other targets). Figure done using Biorender

The effectiveness of cMSC-conditioned medium is appealing in a clinical perspective as MSC will continuously produce and diffuse its secretome upon injection in the body until their elimination. The Fig. 8 presents a synthetic, simplified overview of the conditioning in vitro and its potential actors as suggested from this study.

### cMSC show therapeutic efficacy in vivo

The NSG-MG model was previously developed to closely resemble the pathophysiology of the human MG disease. Although the triggering events responsible for this autoimmune neuromuscular disorder are unknown and cannot be translated into a model yet, the other components are present in the NSG-MG model through the grafting of thymus remnants containing T and B cells and the thymic inflammatory micro-environment. In this model, components of immune system are actively transferred, and the multiple immune dysregulations may be counteracted by the multiple immunomodulatory mechanisms brought by MSC [34]. The composite clinical scores documented significant differences brought by the injection of cMSC grown using hPL. The cMSC treatment did not stop the disease but slowed its progression over the weeks of follow-up. This confirmed and extended the observations obtained previously using MSC of research grade grown in FBS [34]. Such a confirmation is important as it underlines the robustness of the effect in conditions close to clinical applicability.

### Limitations of the study

A limitation of the study is the absence of a systematic assessment of IFN-γ-activated cells across all experimental sections. The study primarily focused on mechanistic insights into PBMC conditioning. IFN- γ was utilized as a control in major steps, including RNASeq studies, qPCR validations, flow and mass cytometry phenotyping, and functional inhibition of proliferation in vitro. However, the study did not aim to delineate the secretome produced by cells after IFN- γ treatment or evaluate the efficacy of IFN-γ-activated cells in vivo. It was, nevertheless, crucial to validate the quality and responsiveness of cell batches using a well-established stimulus like IFN-γ activation.

The search of the mechanisms of actions deployed by PBMC or MSC to exert their respective effects led to propose candidate genes and proteins. Using the proposed readouts, it will be of great interest to validate or infirm the pathways using either pharmacological molecules or molecular activators or inhibitors (i.e. siRNAs…). We started such a study, in exploring the potential place of CD26 in the inhibition of activated T cells, using the pharmacological inhibitor saxagliptin. However, several pathways exist, that deserve explorations. Future researches should also explore alternative pro-inflammatory cocktails of molecules to further elucidate the mechanisms of PBMC conditioning, and finally replace the use of PBMC, which introduce variabilities. This can be achieved by employing recombinant conditioning molecules identified in this study by secretome and RNASeq analysis. Such research may still improve efficacy of conditioning and is currently underway. Similarly, our new set of potentially immunomodulatory proteins will deserve individual assessement in future studies.

## Conclusion

In conclusion, by integrating transcriptomic, proteomic, phenotypic, and functional analyses, we obtained a detailed description of MSC conditioning by PBMC. This study demonstrates that PBMC conditioning modulates the expression of specific genes highlighting key immune mediators such as *CCL2*, *CCL11*,* DPP4*,* ICAM1*,* IL6*,* PDCD1LG2*,* TNFRSF11B*,* TNIP1*,* TNIP3* and *ZC3H12A*, that it induces phenotypic changes encompassing CD26 (*DPP4*), CD54 (*ICAM1*) and CD273 (*PDCD1LG2*), and leads to the production of proteins involved in the inhibition of proliferation and the modulation of immune cell subsets. Our findings highlight that PBMC conditioning and IFN-γ treatment operate through distinct mechanisms. We propose robust signatures to monitor MSC status throughout their production for clinical applications and contemplate the use of such conditioning for immunomodulation, especially in autoimmune diseases.

## Supplementary Information

Below is the link to the electronic supplementary material.

Supplementary Material 1

Supplementary Material 2

Supplementary Material 3

Supplementary Material 4

Supplementary Material 5

## Data Availability

The sequencing data (RNASeq analysis) supporting the results of this study are available in the European Nucleotide Archive (ENA) at EMBL-EBI under accession number PRJEB77871 (ERP162200). Additional data and materials can be made available upon reasonable request from corresponding author.

## Abbreviations

AChR: Acetylcholine receptor
CFSE: Carboxy fluorescein succinimidyl ester
CyTOF: Cytometry by time of flight
ECM: Extracellular matrix
FBS: Fetal bovine serum
hPL: human Platelet lysate
IDO-1: Indoleamine-2,3 dioxygenase
IFN: Interferon
MFI: Mean fluorescence intensity
MG: Myasthenia gravis
MSC: Mesenchymal stromal cells
NSG: NOD SCID Gamma
PAF: Paraformaldehyde
PBMC: Peripheral blood mononuclear cells
PCA: Principal component analysis
PTGS2: Prostaglandin E2 synthase
RT: Room temperature
SB: Staining buffer
