# Supplementary figures and images for: Mesenchymal stromal cells conditioned by peripheral blood mononuclear cells exert enhanced immunomodulation capacities and alleviate a model of Myasthenia Gravis
Source: Stem Cell Res Ther. 2025 Aug 8;16:437. doi: 10.1186/s13287-025-04534-9 (PMC12333171; doi:10.1186/s13287-025-04534-9)

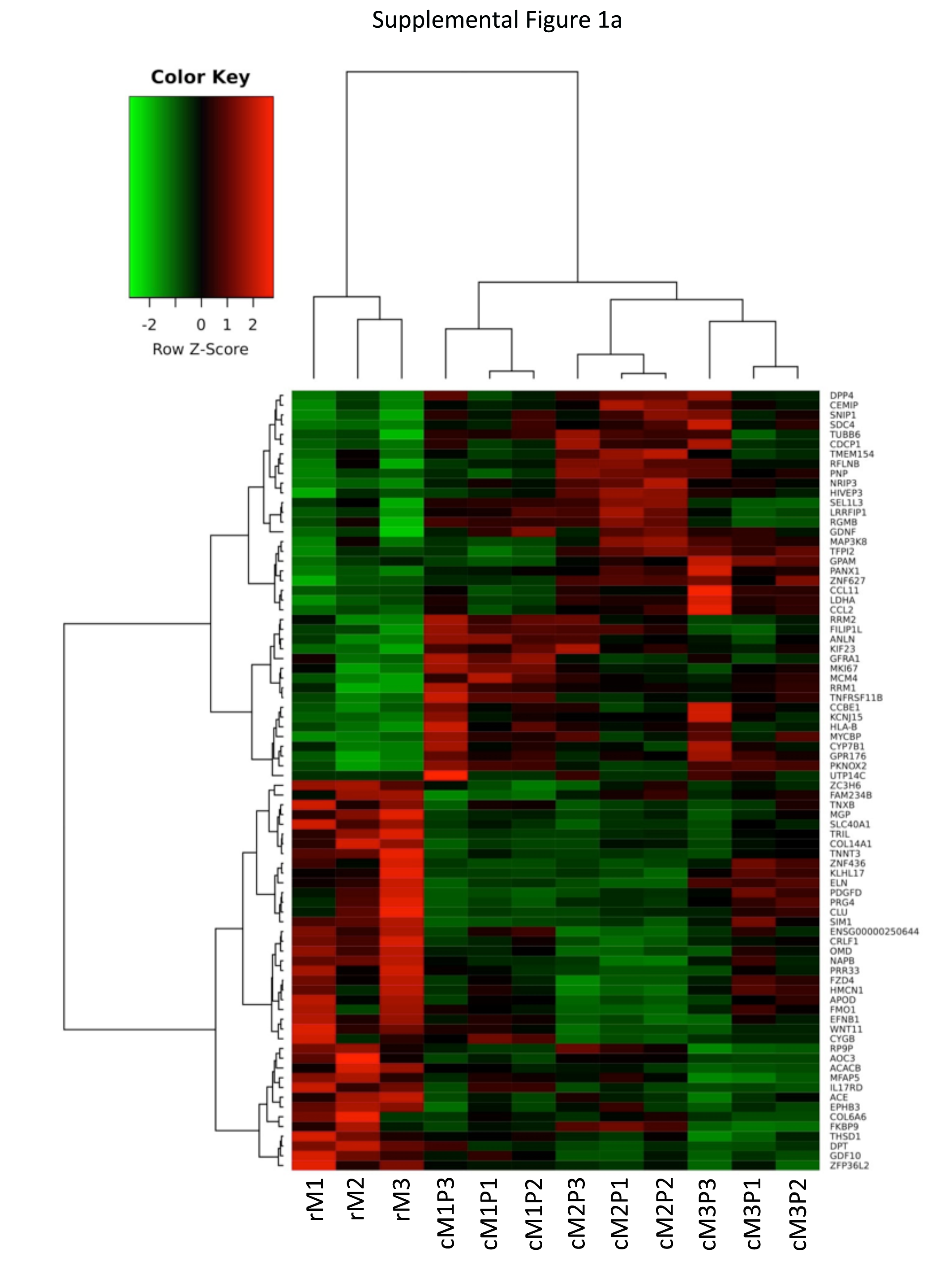

Supplement: Supplementary file 1 — Supplementary Material 1 [file 13287_2025_4534_MOESM1_ESM.tif]

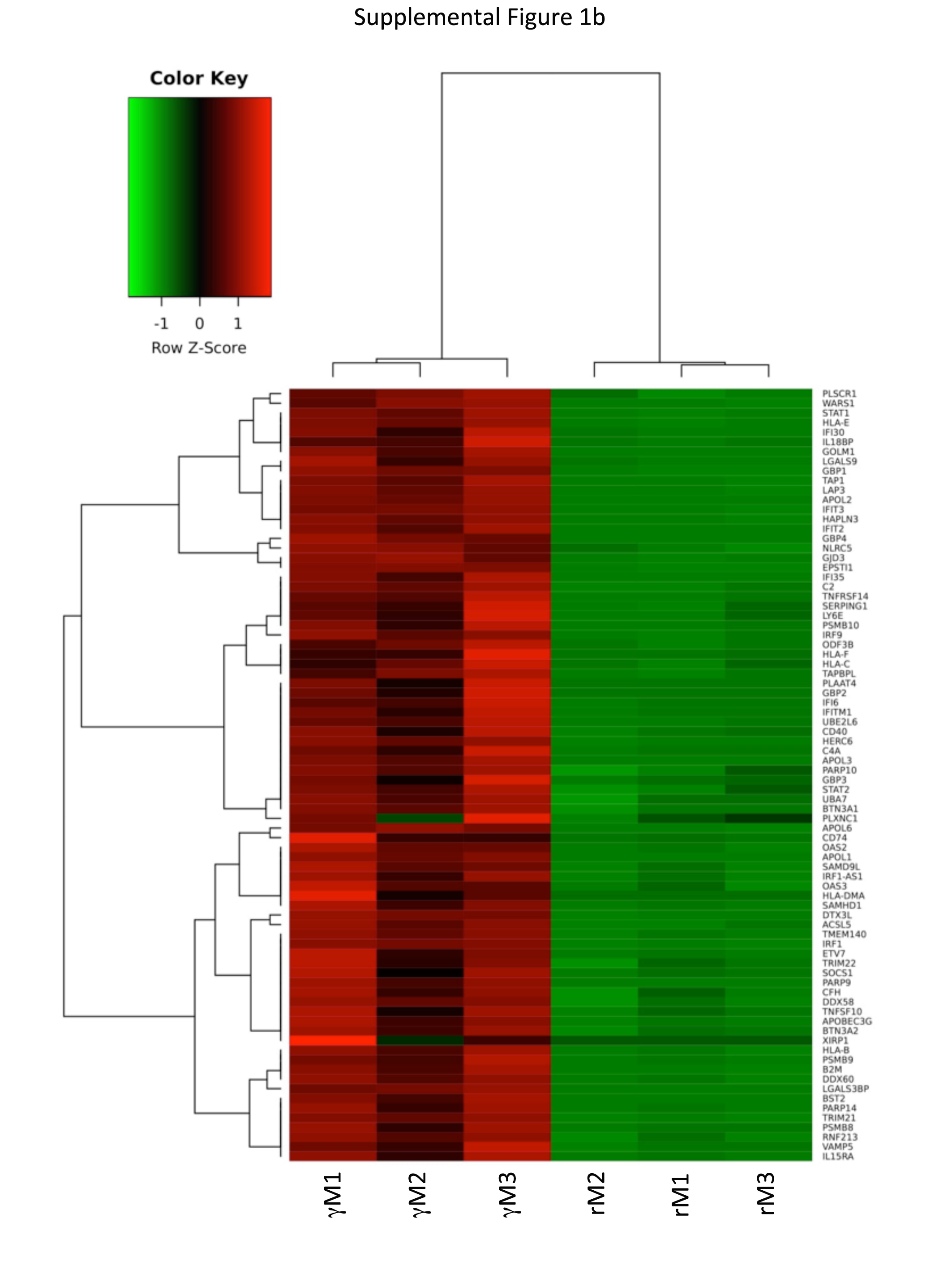

Supplement: Supplementary file 2 — Supplementary Material 2 [file 13287_2025_4534_MOESM2_ESM.tif]

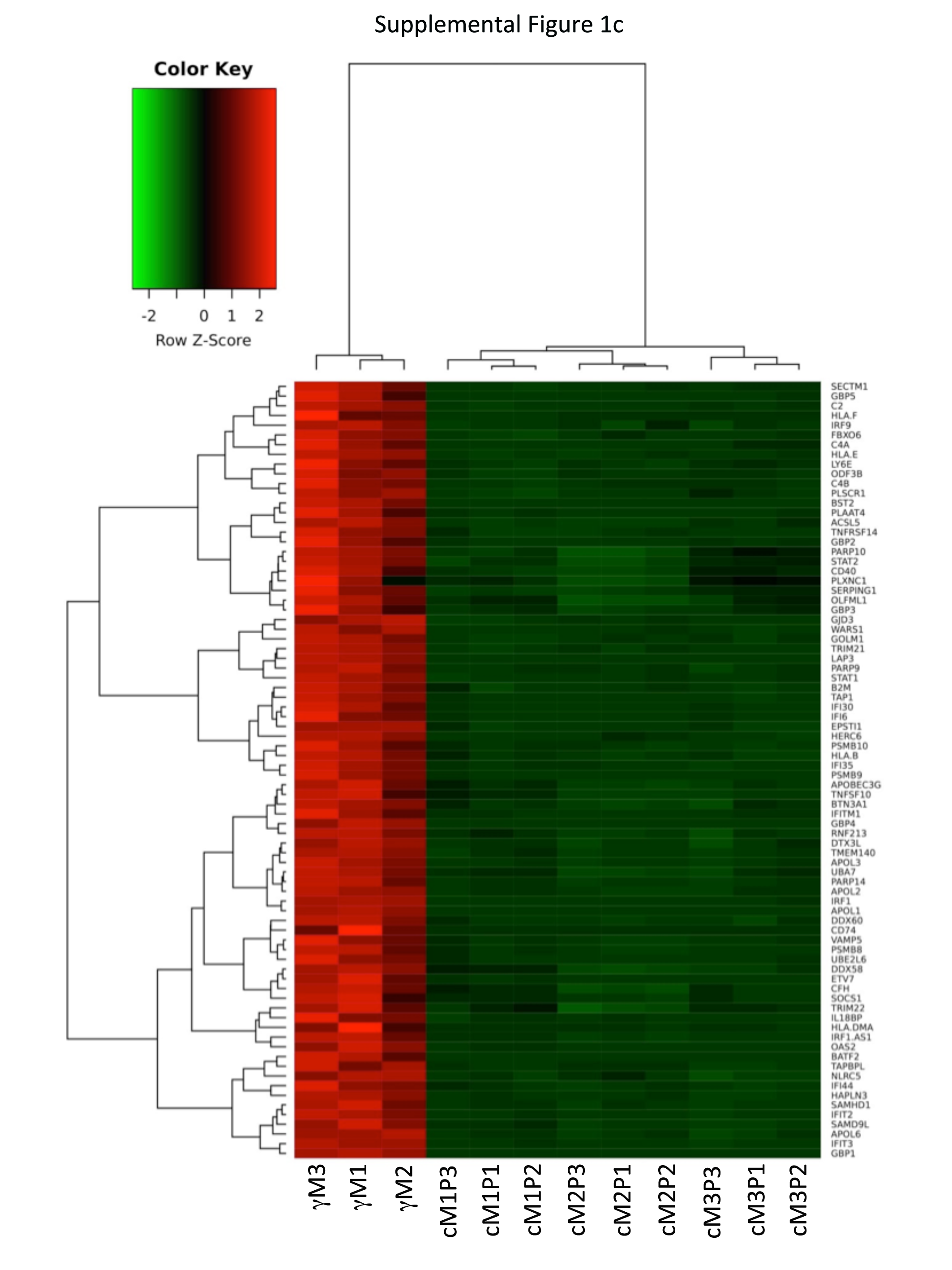

Supplement: Supplementary file 3 — Supplementary Material 3 [file 13287_2025_4534_MOESM3_ESM.tif]
