# Supplementary material for: Mesenchymal stromal cells conditioned by peripheral blood mononuclear cells exert enhanced immunomodulation capacities and alleviate a model of Myasthenia Gravis
Source: Stem Cell Res Ther. 2025 Aug 8;16:437. doi: 10.1186/s13287-025-04534-9 (PMC12333171; doi:10.1186/s13287-025-04534-9)
Supplement: Supplementary file 4 — Supplementary Material 4 [file 13287_2025_4534_MOESM4_ESM.docx]

**Supplemental information titles and legends**

The Supplemental informations contain:

Supplemental Figure 1, consisting in 3 large heatmaps (S1a, S1b, S1c)

Supplemental tables 1-4 (Table S 1, Table S 2, Table S 3, Table S 4) in Word format.

***Supplemental Figure S1a, S1b, S1c***

Heat maps representing the top 80 DEG between rMSC, cMSC and γMSC. Gene-level quantification was done with RSEM (v1.3.1). Raw counts were normalized, genes with low counts were discarded. Analysis for differential expression between conditions, accounting for variations due to batch effect (Batch + Condition), was done fitting to a negative binomial generalized model implemented in the DESeq2 algorithm (R package, version 1.26.0). Genes with adjusted pvalue < 0.05 were regarded as differentially expressed genes. Hierarchical clustering of the top 80 most significant differentially expressed genes was performed on the normalized matrix. Distance between features was measured by (1 - Spearman correlation coefficient) and clustering was performed using the Ward.D2 method. In Figure S1a, resting M1, M2 and M3 are compared with M1, M2 and M3 upon conditioning by P1, P2 and P3 (n = 9 combinations). In Figure S1b, resting M1, M2 and M3 are compared with M1, M2 and M3 upon priming by γIFN. In Figure S1c, M1, M2 and M3 upon priming by γIFN are compared with M1, M2 and M3 upon conditioning by P1, P2 and P3 (n = 9 combinations). These maps display the names of the genes as well as the extent of the expression from low (green) to red (high).

***Table S 1. Antibodies used for MSC characterization by flow or mass cytometry.***

***Table S 2. List of primers used for RT-qPCR experiments.***

***Table S 3. List of the 45 top genes upregulated by PBMC conditioning or IFN-γ priming.***

***Table S 4. Percentages of human CD45^+^ cells contained in NSG-MG mice blood.***
