# Supplementary material for: Mesenchymal stromal cells conditioned by peripheral blood mononuclear cells exert enhanced immunomodulation capacities and alleviate a model of Myasthenia Gravis
Source: Stem Cell Res Ther. 2025 Aug 8;16:437. doi: 10.1186/s13287-025-04534-9 (PMC12333171; doi:10.1186/s13287-025-04534-9)
Supplement: Supplementary file 5 — Supplementary Material 5 [file 13287_2025_4534_MOESM5_ESM.docx]

**Table S 1. Antibodies used for flow and mass cytometry MSC characterization.**

*: antibodies custom-labelled according to manufacturer instructions.

|  | Flow Cytometry | | Mass Cytometry | | |
| --- | --- | --- | --- | --- | --- |
| Antibody | Clone | Supplier | Clone | Metal tag | Supplier |
| CD13 | WM15 | BD | - | - | - |
| CD26 | BA5b | Biolegend | BA5b | 151 Eu | Biolegend * |
| CD34 | 563 | BD | 581 | 166 Er | Fluidigm |
| CD45 | HI30 | BD | HI30 | 89 Y | Fluidigm |
| CD47 | B6H12 | BD | - | - | - |
| CD49a | SR84 | BD | TS2/7 | 163 Dy | Fluidigm |
| CD49c | C3 II.1 | BD | ASC-1 | 161 Dy | Fluidigm |
| CD49e | IIA1 | BD | IIA1 | 176 Yb | BD * |
| CD54 | HA58 | BD | HA58 | 170 Er | Fluidigm |
| CD55 | IA10 | BD | JS11 | 148 Nd | Fluidigm |
| CD59 | H19 | BD | H19 | 173 Yb | Fluidigm |
| CD61 | VIPL2 | BD | VI-PL2 | 209 Bi | Fluidigm |
| CD73 | AD2 | BD | AD2 | 168 Er | Fluidigm |
| CD90 | 5E10 | Dako | 5E10 | 145 Nd, 147 Sm, 164 Dy | BD * |
| CD90 | - | - | Thy-1A1 | 150 Nd, 155 Gd | R&D * |
| CD90 | - | - | F15-42-1 | 149 Sm | Thermofisher * |
| CD105 | SN6 | eBiosciences | 266 | 152 Sm | BD * |
| CD112 | TX31 | Biolegend | TX31 | 169 Tm | Ozyme * |
| CD120b | 3G7A02 | Biolegend | 3B7A02 | 171 Yb | Biolegend * |
| CD140a | αR1 | BD | D13C6 | 160 Gd | Fluidigm |
| CD140b | 28D4 | BD | 18A2 | 156 Gd | Fluidigm |
| CD155 | SKIL4 | Biolegend | 1C6 | 165 Ho | Ozyme * |
| CD172a/b | SE5A5 | Biolegend | SE5A5 | 175 Lu | Fluidigm |
| CD194 | 1G1 | BD | L294H4 | 158 Gd | Biolegend * |
| CD273 | 24F.10C12 | Biolegend | 24F.10C12 | 172 Yb | Fluidigm |
| CD274 | MIH3 | Biolegend | 29E.2A3 | 159 Tb | Fluidigm |
| CD317 | R538E | Biolegend | RS38E | 144 Nd | Biolegend * |
| CD318 | CUB1 | Biolegend | - | - | - |
| HLA-ABC | G46-2.6 | BD | W6/32 | 141 Pr | Fluidigm |
| HLA-DR | L243 | Biolegend | L243 | 174 Yb | Fluidigm |
| IDO1 | - | - | 703808 | 154 Sm | R&D * |
| PTGS2 | - | - | Polyclonal | 143 Nd | R&D * |
| Ki-67 | - | - | B56 | 162 Dy | Fluidigm |

| Gene | Left Primer | Right Primer | Product size (bp) | Elongation time (sec) | Annealing T^a^ (°C) |
| --- | --- | --- | --- | --- | --- |
| CCL2 | AGCAGCAAGTGTCCCAAAGA | TCTGGGGAAAGCTAGGGGAA | 195 | 14 | 60 |
| CCL8 | GGGACTTGCTCAGCCAGATT | CATCTCTCCTTGGGGTCAGC | 186 | 18 | 60 |
| CCL11 | CCCAGAAACCACCACCTCTC | TGCCACTGGTGATTCTCCTG | 216 | 14 | 60 |
| CD74 | AGACAGATCCCCGTTCCTGA | GGGAAAGGGAAGAGAGTGGC | 211 | 18 | 60 |
| CILP | AGGCTGGGGAGTACTTTTGC | AGTCTTAACAGGGCAGCGTC | 200 | 14 | 62 |
| CXCL9 | TGAGAAAGGGTCGCTGTTCC | GCTGACCTGTTTCTCCCACT | 206 | 14 | 62 |
| CXCL10 | CTGCCTCTCCCATCACTTCC | GCAGGGTCAGAACATCCACT | 227 | 18 | 60 |
| CXCL11 | CTCCTTCCAAGAAGAGCAGCA | GCGTCCTCTTTTGAACATGGG | 156 | 14 | 62 |
| DPP4 | GCCACTTACCTTGCAAGCAC | CCGATCCCAGGACCATTGAG | 238 | 14 | 60 |
| HLA-DRA | AGACAAGTTCACCCCACCAG | AGCATCAAACTCCCAGTGCT | 220 | 18 | 60 |
| ICAM1 | TTGGGCACTGCTGTCTACTG | GAAGTCCCAGCCCCATTTGA | 226 | 14 | 62 |
| IDO1 | ACATGCTGCTCAGTTCCTCC | CTGGCTTGCAGGAATCAGGA | 223 | 14 | 60 |
| IL6 | TACCCCCAGGAGAAGATTCC | GCCATCTTTGGAAGGTTCAG | 199 | 14 | 62 |
| LGALS1 | AAACCTGGAGAGTGCCTTCG | GGAAGGGAAAGACAGCCTCC | 205 | 14 | 62 |
| PDCD1LG2 | GCAATGTGACCCTGGAATGC | GTCCTTCGTCCCTCACTTGG | 189 | 14 | 60 |
| PTGS2 | CATCCCCTTCTGCCTGACAC | GCTCTGGTCAATGGAAGCCT | 204 | 18 | 60 |
| TGFB1 | GGGACTATCCACCTGCAAGA | CCTCCTTGGCGTAGTAGTCG | 239 | 14 | 62 |
| TNFAIP3 | TCGACAGAAACATCCAGGCC | AACAGCGCCTTCCTCAGTAC | 175 | 14 | 62 |
| TNFRSF11B | TGAACAACTTGCTGTGCTGC | ACGGTCTTCCACTTTGCTGT | 181 | 14 | 62 |
| TNIP1 | TCAAAACCTCCCGGAAGTGG | GCAAGGCTACTGTGAGTGGT | 220 | 14 | 60 |
| TNIP3 | ACCAGCAATGGGATCAGCAA | TCTCTCTGCCTGTCGTCCTT | 154 | 18 | 60 |
| ZC3H12A | TTGTGAAGCTGGCCTACGAG | TGAGTGGCTTCTTACGCAGG | 207 | 18 | 60 |

**Table S 2. List of primers used for RT-qPCR**

**Table S 3: List of the 45 top genes upregulated by PBMC conditioning or IFNγ priming**

**Table S 4. Percentages of human CD45^+^ cells contained in NSG-MG mice blood.**

NA: not available (found dead or previous euthanasia)

| First cohort | | | | |
| --- | --- | --- | --- | --- |
| Treatment | Mouse ID | Week 2 | Week 4 | Euthanasia |
| NaCl | 1 | 3,75 | NA | NA |
|  | 2 | 14,50 | 84,60 | 60,40 |
|  | 3 | 2,30 | 51,40 | 59,10 |
|  | 4 | 38,40 | 82,30 | NA |
| rMSC | 1 | 3,60 | 78,70 | 39,60 |
|  | 2 | 41,50 | 80,80 | 66,10 |
|  | 3 | 17,40 | 85,00 | 69,60 |
|  | 4 | 2,25 | 82,30 | 43,00 |
| cMSC | 1 | 2,20 | 75,20 | 61,90 |
|  | 2 | 30,60 | 75,80 | 43,20 |
|  | 3 | 43,60 | NA | NA |
|  | 4 | 0,40 | 8,90 | 32,40 |

| Second cohort | | | | |
| --- | --- | --- | --- | --- |
| Treatment | Mouse ID | Week 2 | Week 4 | Euthanasia |
| NaCl | 1 | 0,8 | 5 | 15,5 |
|  | 2 | 0,2 | 0,25 | 0,35 |
|  | 3 | 0,15 | 5,8 | 3,6 |
|  | 4 | 0,15 | 0,45 | 15,1 |
|  | 5 | 0,2 | 6,1 | NA |
| rMSC | 1 | 0,15 | 1 | 10,7 |
|  | 2 | 0,5 | 10,5 | 14,9 |
|  | 3 | 0,2 | 10,5 | 21,5 |
|  | 4 | 0,5 | 17 | 18,5 |
|  | 5 | 0,2 | 2,8 | 7,95 |
| cMSC | 1 | 0,5 | 4,1 | 54,5 |
|  | 2 | 0,1 | 0,2 | 8,3 |
|  | 3 | 0,25 | 0,7 | 11,1 |
|  | 4 | 0,6 | 1,9 | 16,8 |
|  | 5 | 1,15 | 0,75 | 4,3 |
